# Coronary Artery Plaque Phenotype and 5-Year Clinical Outcomes in Older Patients with Non-ST Elevation Acute Coronary Syndrome

**DOI:** 10.31083/j.rcm2505168

**Published:** 2024-05-14

**Authors:** Francesca Rubino, Salvatore Brugaletta, Gregory Mills, Graziella Pompei, Roberto Scarsini, Flavio Ribichini, Lorenz Räber, Vijay Kunadian

**Affiliations:** ^1^Translational and Clinical Research Institute, Faculty of Medical Sciences, Newcastle University, NE2 4HH Newcastle upon Tyne, UK; ^2^Division of Cardiology, Department of Medicine, University of Verona, 37126 Verona, Italy; ^3^Hospital Clínic, Cardiovascular Clinic Institute, Institut d'Investigacions Biomèdiques August Pi i Sunyer (IDIBAPS), University of Barcelona, 08036 Barcelona, Spain; ^4^Cardiothoracic Centre, Freeman Hospital, Newcastle Upon Tyne Hospitals NHS Foundation Trust, NE7 7DN Newcastle Upon Tyne, UK; ^5^Cardiovascular Institute, Azienda Ospedaliero-Universitaria di Ferrara, 44124 Cona, FE, Italy; ^6^Department of Cardiology, Bern University Hospital, University of Bern, 3010 Bern, Switzerland

**Keywords:** virtual-histology intravascular ultrasound, non-ST elevation acute coronary syndrome, major adverse cardiovascular events, minimal lumen area, plaque burden, thin-cap fibroatheroma, older patients

## Abstract

**Background:**

Lesions with thin-cap fibroatheroma (TCFA), small luminal area and large plaque burden (PB) have been considered at high risk of cardiovascular events. Older patients were not represented in studies which demonstrated correlation between clinical outcome and plaque characteristics. This study aims to investigate the prognostic role of high-risk plaque characteristics and long-term outcome in older patients presenting with non-ST elevation acute coronary syndrome (NSTEACS).

**Methods:**

This study recruited older patients aged ≥75 years with NSTEACS undergoing virtual-histology intravascular ultrasound (VH-IVUS) imaging from the Improve Clinical Outcomes in high-risk patieNts with acute coronary syndrome (ICON-1). Primary endpoint was the composite of major adverse cardiovascular events (MACE) consisting of all-cause mortality, myocardial infarction (MI), and any revascularisation. Every component of MACE and target vessel failure (TVF) including MI and any revascularisation were considered as secondary endpoints.

**Results:**

Eighty-six patients with 225 vessels undergoing VH-IVUS at baseline completed 5-year clinical follow-up. Patients with minimal lumen area (MLA) ≤4 ${\mathrm{mm}}^{2}$ demonstrated increased risk of MACE (hazard ratio [HR] 2.37, 95% confidence interval [CI] 1.00–5.59, *p* = 0.048) with a worse event-free survival (Log Rank 4.17, *p* = 0.041) than patients with MLA >4 ${\mathrm{mm}}^{2}$. Patients with combination of TCFA, MLA ≤4 ${\mathrm{mm}}^{2}$ and PB ≥70% showed high risk of MI (HR 5.23, 95% CI 1.05–25.9, *p* = 0.043). Lesions with MLA ≤4 ${\mathrm{mm}}^{2}$ had 6-fold risk of TVF (HR 6.16, 95% CI 1.24–30.5, *p* = 0.026).

**Conclusions:**

Small luminal area appears as the major prognostic factor in older patients with NSTEACS at long-term follow-up. Combination of TCFA, MLA ≤4 ${\mathrm{mm}}^{2}$ and PB ≥70% was associated with high risk of MI.

**Clinical Trial Registration:**

NCT01933581.

## 1. Introduction

Ischaemic heart disease is leading cause of death worldwide and one of the most important reasons for loss of health status [1]. In the United States 720,000 patients experience acute coronary syndrome (ACS) every year and more than 50% of them have non-ST-elevation myocardial infarction (NSTEMI) [2]. Patients ≥75 years old represent the 30–40% of all patients with ACS [3].

Numerous studies [4, 5] investigated the pathological and biological basis of atherosclerosis identifying the characteristics of vulnerable plaques. Active inflammation with infiltration of leukocytes such as macrophages promotes plaque progression [4]. Presence of a thin fibrous layer of intimal tissue covering the necrotic core of a lipid-rich fibroatheroma has been recognised as cause of myocardial infarction (MI) and cardiovascular death [4, 5]. These characteristics increase the probability of plaque rupture or erosion and formation of an intraluminal thrombus. Pathogenesis of non-ST elevation acute coronary syndrome (NSTEACS) usually includes a flow-limiting coronary stenosis leading to myocardial ischaemia. On the other hand, ST-elevation myocardial infarction (STEMI) is caused by a total acute coronary thrombosis.

Studies using intravascular imaging techniques including virtual-histology intravascular ultrasound (VH-IVUS), near-infrared spectroscopy and optical coherence tomography (OCT), investigated the correlation between plaque composition and adverse clinical outcomes. Thin-cap fibroatheroma (TCFA), small luminal area and large plaque burden (PB), in particular minimal lumen area (MLA) ≤4 ${\mathrm{mm}}^{2}$ and PB ≥70%, have been demonstrated to be risk factors for cardiovascular events [6, 7, 8]. However, these findings have been evaluated in a population with a median age lower than 65 years old. Life expectancy has grown in recent years with population ageing [9]. The incidence of cardiovascular diseases, especially ischaemic heart disease, increases with age [10]. It is expected that more and more older patients with ischaemic heart disease will need to be treated. There is a gap in literature about the prognostic role of plaque characteristics and prognosis in older patients. Therefore, further data are required to improve the clinical management of older patients.

In previous studies, older patients had larger PB and different plaque composition with greater necrotic core, calcifications, cholesterol crystals and lipid-core than young patients [11, 12]. The Improve Clinical Outcomes in high-risk patieNts with ACS (ICON-1) study demonstrated the association between frailty phenotype and plaque morphology. It explored the correlation between high-risk plaque characteristics and 1-year clinical outcome in older patients with NSTEACS [13]. The impact of high-risk plaque phenotypes on long-term prognosis in older patients is poorly understood. The current study is a sub-analysis of the multicentre, observational and prospective ICON1 study which aims to investigate the long-term prognostic role of high-risk plaque characteristics defined by VH-IVUS in older patients with NSTEACS.

## 2. Materials and Methods

### 2.1 Study Population

This study includes a population assessed with intracoronary imaging from the ICON-1 study. ICON-1 is a multicentre, observational and prospective cohort study that investigated older patients aged ≥75 years with non-ST-elevation acute coronary syndrome (NSTEACS) undergoing invasive coronary angiography [14]. The study was conducted in accordance with the Declaration of Helsinki and was approved by the National Research Ethics Service (12/NE/01600). It was registered with the ClinicalTrials.gov (NCT01933581) and United Kingdom Clinical Research Network (UKCRN; ID 12742). All the patients included agreed to participate in the study and signed informed consent. Older patients with NSTEACS referred to two tertiary cardiac centres, Freeman Hospital, Newcastle upon Tyne and James Cook University Hospital, Middlesbrough, were recruited between November 2012 and December 2015. Inclusion and exclusion criteria have been previously published in the study protocol and they are reported in the **Supplementary Methods **[14]. The study population considered patients with 5-year follow-up data as shown in the study flow chart (**Supplementary Fig. 1**).

### 2.2 Baseline Data Collection

Data were collected at the time of recruitment by members of the research team including the principal investigator, research fellows and nurses. Baseline characteristics included demographic information, clinical scores, medical history, blood tests, procedural data and discharge medical therapy. Fried frailty criteria from the Cardiovascular Health Study, including weight loss, exhaustion, physical inactivity, weakness and slow walking/getting up from chair, were used to assess Frailty status. Each criterion provides one point. A score ≥3 defines frail patients, a score of 1 or 2 pre-frail patients and a score of 0 robust patients [15]. Blood samples were collected at the time of coronary angiography or percutaneous coronary intervention (PCI) for analysis. Increased levels of interleukin-6 (IL-6) have been found in patients with acute coronary syndrome [16, 17, 18, 19]. IL-6 may contribute to coronary plaque instability [20]. Therefore, IL-6 data were analysed in our study. The cut-off of IL-6 ≥5 ng/L has been chosen a priori based on a previous research study which investigated the relationship between IL-6 and mortality in a population with unstable coronary artery disease [21].

### 2.3 Virtual Histology-Intravascular Ultrasound Assessment

Patients underwent VH-IVUS of all three coronary arteries following invasive coronary angiography prior to PCI. Intravascular imaging was performed with a 20 MHz, phased array Eagle Eye Platinum catheter, that was mounted on an R-100 pullback device and connected to either an integrated s5i system or a mobile s5 tower (Philips Volcano, San Diego, CA, USA). Imaging was ECG-gated and acquired at a pullback speed of 0.5 mm/s. Seven patients investigated with a 45 MHz Revolution catheter were excluded to keep consistency of data (**Supplementary Fig. 1**).

Anonymous data were analysed in the Newcastle Angiography/IVUS/optical coherence tomography core laboratory using the Medis QIVUS software, versions 2.2 and 3.0 (Medis medical imaging systems, Leiden, the Netherlands). The detailed description of data analysis has been provided previously [13]. IVUS measurements consisted of cross-sectional areas of external elastic membrane, lumen, plaque and media area, MLA and diameter, PB, percent stenosis, absolute volume and percentage of total plaque volume for each plaque component (fibrous tissue, fibro-fatty tissue, necrotic core, dense calcium). Lesion phenotypes including intimal medial thickening, pathological intimal thickening, fibrotic plaque, fibrocalcific plaque, thick-cap fibroatheroma, calcified thick-cap fibroatheroma, TCFA and calcified TCFA were performed on VH-IVUS according to definitions from the consensus document as described previously [13, 22]. Different plaque phenotypes are described as percentage over the vessel. Data regarding lesions or patients were analysed separately.

### 2.4 Follow-up and Clinical Outcomes

Long term follow-up data was derived from Summary Care Records, National Health Service Digital and tertiary centre hospital electronic records. Summary Care Record is a national electronic summary of important patient information created from general practitioners. Research Ethics Committee approved the 5-year follow up study (REC 12/NE/0160).

The primary endpoint was major adverse cardiovascular events (MACE), a composite of all-cause mortality, any MI, and any revascularisation. The secondary endpoints were individual components of the primary endpoint and target vessel failure (TVF) including MI and any revascularisation. Vessel-oriented events were considered when images from coronary angiography were available to identify a specific vessel. MI with non-obstructive coronary arteries was excluded from the vessel-oriented endpoint. In addition, the association between plaque characteristics and IL-6 was explored as part of secondary endpoints.

MI was defined as a primary diagnosis of non–ST- segment elevation MI or ST-segment elevation MI according to the 4th universal definition of MI [23]. Any revascularisation included percutaneous or surgical revascularization of the coronary arteries.

### 2.5 Statistical Analysis

Categorical data are presented as absolute numbers and percentages. Comparison between categorical variables were analysed using Fisher’s exact test or the Pearson’s chi-square test, as appropriate. Continuous data are described as mean plus or minus standard deviation (± SD) or median and interquartile range (IQR) according to normality of distribution. Comparison between normally distributed continuous variables were performed using Student’s *t* test. Mann-Whitney U test was used for comparison between non-normally distributed continuous data. Survival analysis was computed using Kaplan-Meier curves explaining event-free survival. Differences between the two groups were evaluated using Log-Rank test. The association between plaque characteristics and outcomes was estimated using Cox-regression univariable and multivariable analysis. The hazard ratio (HR) with 95% confidence interval (CI) was considered. Adjusted hazard ratio (aHR) in multivariable analysis was provided only for MACE given small numbers of every component of primary endpoint to estimate a model. Association between plaque phenotype and IL-6 was investigated using logistic regression model, presented as odds ratio (OR) with 95% CI. Statistically significant results were considered when *p*-value was ≤0.05. The follow-up period was censored at 5 years and time-to-first events was used for data analysis. Statistical Package for the Social Sciences (SPSS V.29, IBM Corporation, Armonk, NY, USA) was used for all statistical analysis.

## 3. Results

### 3.1 Recruitment and Baseline Characteristics

Eighty-six patients with 225 vessels, analysed with VH-IVUS, completed 5-year follow-up (median 5 [IQR 3–5] years) (**Supplementary Fig. 1**). The median age was 80.6 (IQR 78.2–83.2) years, and 29 (33.7%) patients were female (**Supplementary Table 1**). Frailty status including frail and pre-frail patients was found in 61 (71.8%) patients (**Supplementary Table 1**). The mean Global Registry of Acute Coronary Events (GRACE) score 2.0 was 128.8 (±18.4) (**Supplementary Table 1**). Sixty-eight (79.1%) patients presented non-ST elevation myocardial infarction (NSTEMI) (**Supplementary Table 1**). Fifty-three (61.6%) patients had arterial hypertension and 44 (51.2%) patients had hyperlipidaemia. The other comorbidities are described in **Supplementary Table 1**.

The median MLA was 5.3 (IQR 3.5–8.0) ${\mathrm{mm}}^{2}$ and the median PB 65.4 (IQR 55.0–73.7) % (**Supplementary Table 2**). Fifty-six (65.1%) patients had TCFA lesions, and 51 (59.3%) patients had lesions with MLA ≤4 ${\mathrm{mm}}^{2}$. PB ≥70% was found in 50 (58.1%) patients. The combination of lesions with TCFA and MLA ≤4 ${\mathrm{mm}}^{2}$ was present in 36 (42%) patients. Thirty-nine (45.3%) patients showed lesions with TCFA and PB ≥70% and 33 (38.4%) patients demonstrated coexistence of all three high-risk plaque characteristics. Baseline characteristics of patients and vessels according to high-risk plaque features are described in **Supplementary Tables 3,4**.

### 3.2 Primary Outcome

**Fig. 1.**
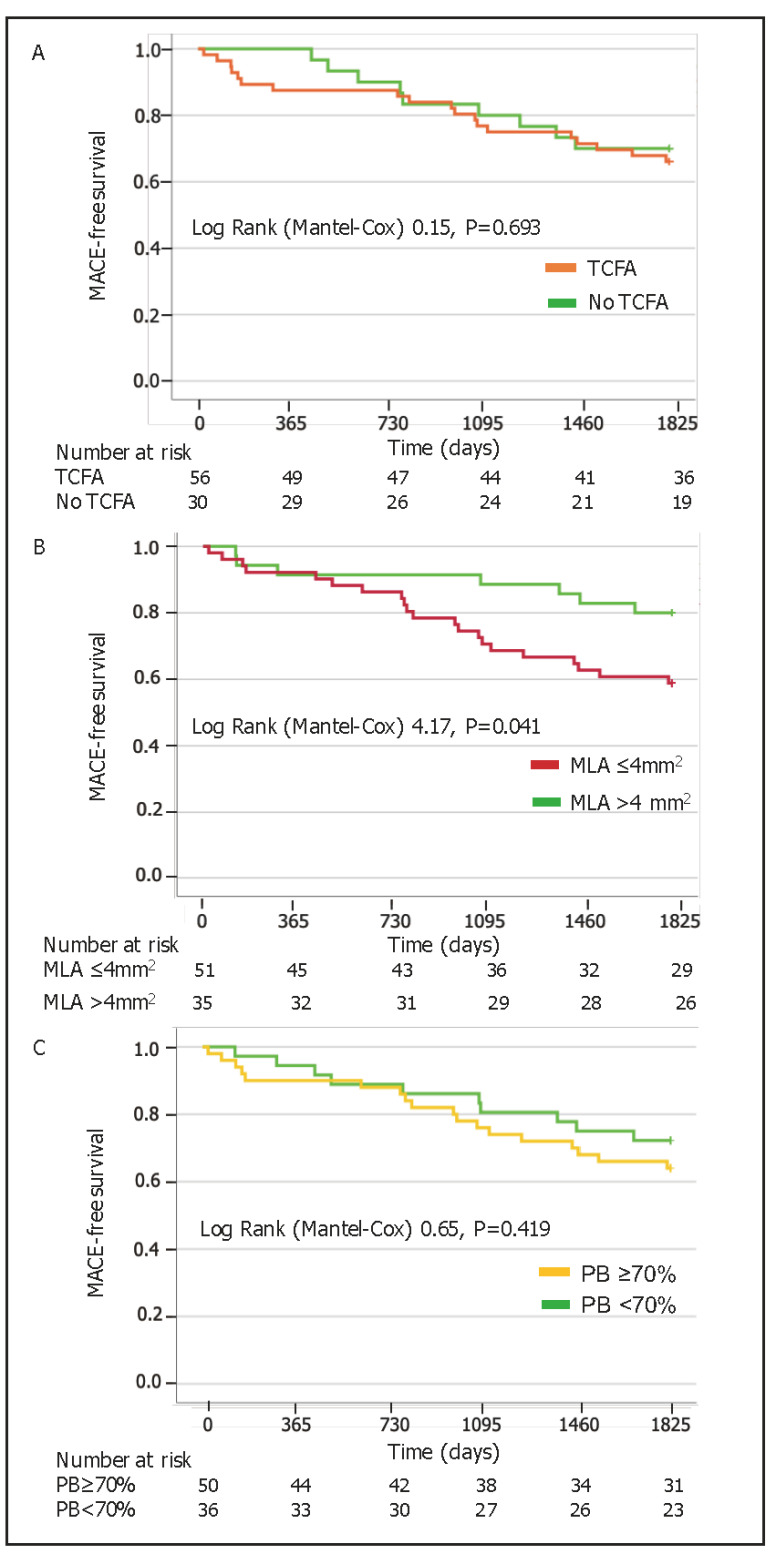
**Kaplan-Meier survival analysis**. MACE-free survival in patients with TCFA (A), MLA ≤4 ${\mathrm{mm}}^{2}$ (B) and PB ≥70% (C). MACE, major adverse cardiovascular events; MLA, minimal lumen area; PB, plaque burden; TCFA, thin-cap fibroatheroma.

**Fig. 2.**
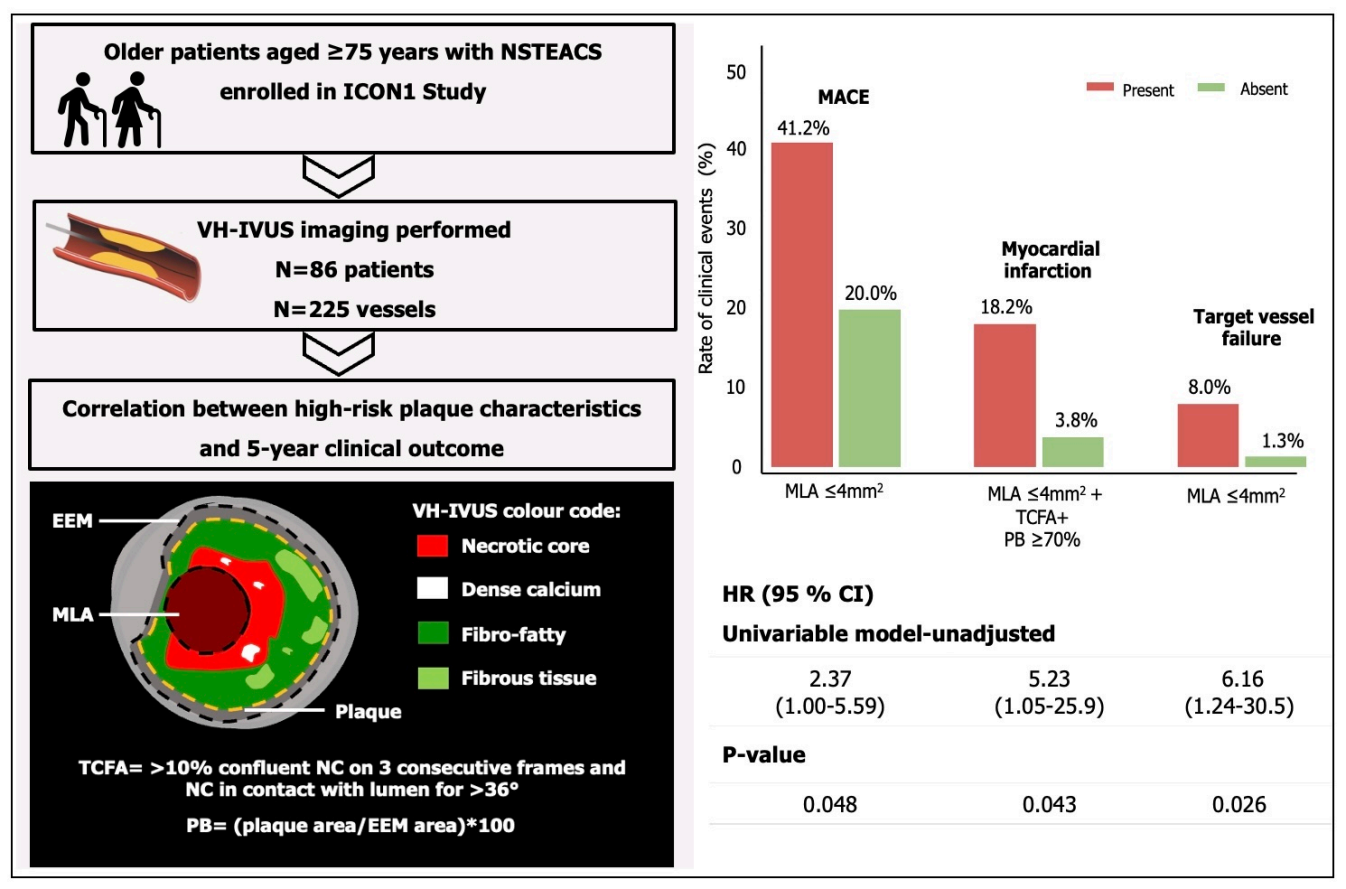
**Brief explanation of study design. Schematic representation of plaque characteristics analysed with VH-IVUS**. Association between high-risk plaque characteristics and adverse clinical events. CI, confidence interval; EEM, external elastic membrane; HR, hazard ratio; ICON1, Improve Clinical Outcomes in high-risk patieNts with acute coronary syndrome; MACE, major adverse cardiovascular events; MLA, minimal lumen area; NC, necrotic core; NSTEACS, non-ST elevation acute coronary syndrome; PB, plaque burden; TCFA, thin-cap fibroatheroma; VH-IVUS, virtual-histology intravascular ultrasound.

The composite endpoint occurred in 28 (32.6%) patients during the follow-up period (Table 1). Patients with MLA ≤4 ${\mathrm{mm}}^{2}$ showed higher incidence of MACE (41.2% *vs.* 20.0%, *p* = 0.040) (Table 1). No other statistically significant differences were found between groups of patients with and without high-risk plaques characteristics (Table 1). Kaplan-Meier curves survival analysis revealed difference in terms of MACE among patients with MLA ≤4 ${\mathrm{mm}}^{2}$ (Log Rank 4.17, *p* = 0.041) (Fig. 1B). MACE-free survival was similar between patients with other plaque characteristics (Fig. 1A,C and **Supplementary Fig. 2**). Patients with MLA ≤4 ${\mathrm{mm}}^{2}$ demonstrated 2-fold risk of MACE versus patients with MLA >4 ${\mathrm{mm}}^{2}$ (HR 2.37, 95% CI 1.00–5.59, *p* = 0.048) (Table 2 and Fig. 2).

**Table 1.** **5-year patients-oriented clinical outcomes and vessels-oriented clinical outcomes stratified by high-risk plaque characteristics**.

Patients-oriented outcomes	Total population (patients n = 86)								
MACE, n (%)	28 (32.6)								
All-cause death, n (%)	20 (23.3)								
MI, n (%)	8 (9.3)								
Any revascularisation, n (%)	9 (10.5)								
High-risk plaque characteristics									
	TCFA	No TCFA	*p*-value	MLA ≤4 ${\mathrm{mm}}^{2}$	MLA >4 ${\mathrm{mm}}^{2}$	*p*-value	PB ≥70%	PB <70%	*p*-value
	(n = 56)	(n = 30)		(n = 51)	(n = 35)		(n = 50)	(n = 36)	
MACE, n (%)	19 (33.9)	9 (30.0)	0.711	21 (41.2)	7 (20.0)	0.040	18 (36.0)	10 (27.8)	0.422
All-cause death, n (%)	13 (23.2)	7 (23.3)	0.990	15 (29.4)	5 (14.3)	0.125	13 (26.0)	7 (19.4)	0.478
MI, n (%)	7 (12.5)	1 (3.3)	0.252	7 (13.7)	1 (2.9)	0.134	6 (12.0)	2 (5.6)	0.459
Any revascularisation, n (%)	6 (10.7)	3 (10.0)	1.00	7 (13.7)	2 (5.7)	0.300	6 (12.0)	3 (8.3)	0.729
	TCFA + MLA ≤4 ${\mathrm{mm}}^{2}$	No TCFA + MLA >4 ${\mathrm{mm}}^{2}$	*p*-value	TCFA + PB ≥70%	No TCFA + PB <70%	*p*-value	TCFA + MLA ≤4 ${\mathrm{mm}}^{2}$ + PB ≥70%	No TCFA + MLA >4 ${\mathrm{mm}}^{2}$ + PB <70%	*p*-value
	(n = 36)	(n = 50)		(n = 39)	(n = 47)		(n = 33)	(n = 53)	
MACE, n (%)	13 (36.1)	15 (30.0)	0.551	15 (38.5)	13 (27.7)	0.287	13 (39.4)	15 (28.3)	0.286
All-cause death, n (%)	9 (25.0)	11 (22.0)	0.745	11 (28.2)	9 (19.1)	0.322	9 (27.3)	11 (20.8)	0.487
MI, n (%)	6 (16.7)	2 (4.0)	0.064	5 (12.8)	3 (6.4)	0.133	6 (18.2)	2 (3.8)	0.050
Any revascularisation, n (%)	4 (11.1)	5 (10.0)	1.00	4 (10.3)	5 (10.6)	1.00	4 (12.1)	5 (9.4)	0.728
Vessel-oriented outcomes	Total population (vessels n = 225)								
TVF, n (%)	8 (3.6)								
High-risk plaque characteristics									
	TCFA	No TCFA	*p*-value	MLA ≤4 ${\mathrm{mm}}^{2}$	MLA >4 ${\mathrm{mm}}^{2}$	*p*-value	PB ≥70%	PB <70%	*p*-value
	(n = 81)	(n = 144)		(n = 75)	(n = 150)		(n = 80)	(n = 145)	
TVF, n (%)	4 (4.9)	4 (2.8)	0.463	6 (8.0)	2 (1.3)	0.018	5 (6.3)	3 (2.1)	0.136
	TCFA + MLA ≤4 ${\mathrm{mm}}^{2}$	No TCFA + MLA >4 ${\mathrm{mm}}^{2}$	*p*-value	TCFA + PB ≥70%	No TCFA + PB <70%	*p*-value	TCFA + MLA ≤4 ${\mathrm{mm}}^{2}$ + PB ≥70%	No TCFA + MLA >4 ${\mathrm{mm}}^{2}$ + PB <70%	*p*-value
	(n = 34)	(n = 191)		(n = 47)	(n = 178)		(n = 31)	(n = 194)	
TVF, n (%)	3 (8.8)	5 (2.6)	0.103	3 (6.4)	5 (2.8)	0.369	3 (9.7)	5 (2.6)	0.082

MACE, major adverse cardiovascular events; MI, myocardial infarction; MLA, minimal lumen area; PB, plaque burden; TCFA, thin-cap fibroatheroma; TVF, target vessel failure.

**Table 2.** **Cox regression analysis of patients oriented and vessels-oriented clinical outcomes**.

MACE			
		HR (95% CI)	*p*-value
TCFA		1.17 (0.53–2.59)	0.694
Univariable model-unadjusted			
Multivariable model-adjusted (age*)		1.12 (0.50–2.47)	0.779
MLA ≤4 ${\mathrm{mm}}^{2}$		2.37 (1.00–5.59)	0.048
Univariable model-unadjusted			
Multivariable model-adjusted (age*)		2.27 (0.96–5.36)	0.060
PB ≥70%		1.37 (0.63–2.97)	0.421
Univariable model-unadjusted			
Multivariable model-adjusted (age*)		1.27 (0.58–2.77)	0.539
TCFA + MLA ≤4 ${\mathrm{mm}}^{2}$		1.26 (0.60–2.65)	0.541
Univariable model-unadjusted			
Multivariable model-adjusted (age*)		1.16 (0.55–2.44)	0.694
TCFA + PB ≥70%		1.49 (0.71–3.0)	0.287
Univariable model-unadjusted			
Multivariable model-adjusted (age*)		1.34 (0.63–2.83)	0.442
TCFA + MLA ≤4 ${\mathrm{mm}}^{2}$ + PB ≥70%		1.50 (0.71–3.16)	0.281
Univariable model-unadjusted			
Multivariable model-adjusted (age*)		1.36 (0.64–2.87)	0.416
All-cause death†			
	TCFA	0.98 (0.39–2.47)	0.981
	MLA ≤4 ${\mathrm{mm}}^{2}$	2.31 (0.83–6.35)	0.105
	PB ≥70%	1.41 (0.56–3.54)	0.459
	TCFA + MLA ≤4 ${\mathrm{mm}}^{2}$	1.17 (0.48–2.82)	0.726
	TCFA + PB ≥70%	1.57 (0.65–3.80)	0.312
	TCFA + MLA ≤4 ${\mathrm{mm}}^{2}$ + PB ≥70%	1.38 (0.57–3.33)	0.472
Myocardial infarction†			
	TCFA	3.86 (0.47–31.4)	0.206
	MLA ≤4 ${\mathrm{mm}}^{2}$	5.42 (0.66–44.1)	0.114
	PB ≥70%	2.30 (0.46–11.4)	0.306
	TCFA + MLA ≤4 ${\mathrm{mm}}^{2}$	4.42 (0.89–21.9)	0.068
	TCFA + PB ≥70%	3.96 (0.79–19.6)	0.092
	TCFA + MLA ≤4 ${\mathrm{mm}}^{2}$ + PB ≥70%	5.23 (1.05–25.9)	0.043
Any revascularisation†			
	TCFA	1.07 (0.26–4.29)	0.920
	MLA ≤4 ${\mathrm{mm}}^{2}$	3.15 (0.65–15.2)	0.152
	PB ≥70%	1.63 (0.41–6.54)	0.486
	TCFA + MLA ≤4 ${\mathrm{mm}}^{2}$	1.18 (0.31–4.40)	0.801
	TCFA + PB ≥70%	1.10 (0.29–4.12)	0.879
	TCFA + MLA ≤4 ${\mathrm{mm}}^{2}$ + PB ≥70%	1.45 (0.39–5.41)	0.576
TVF†			
	TCFA	1.81 (0.45–7.26)	0.398
	MLA ≤4 ${\mathrm{mm}}^{2}$	6.16 (1.24–30.5)	0.026
	PB ≥70%	3.09 (0.74–12.9)	0.122
	TCFA+ MLA ≤4 ${\mathrm{mm}}^{2}$	3.52 (0.84–14.7)	0.085
	TCFA+ PB ≥70%	2.33 (0.55–9.78)	0.245
	TCFA+ MLA ≤4 ${\mathrm{mm}}^{2}$ + PB ≥70%	3.94 (0.94–16.5)	0.060

*Univariable model unadjusted for age: HR 1.10, 95% CI 1.00–1.21, *p* = 0.031. †Univariable model unadjusted. CI, confidence interval; HR, hazard ratio; MACE, major adverse cardiovascular events; MLA, minimal lumen area; PB, plaque burden; TCFA, thin-cap fibroatheroma; TVF, target vessel failure.

### 3.3 Secondary Endpoints

#### 3.3.1 Patients-Level Analysis

**Fig. 3.**
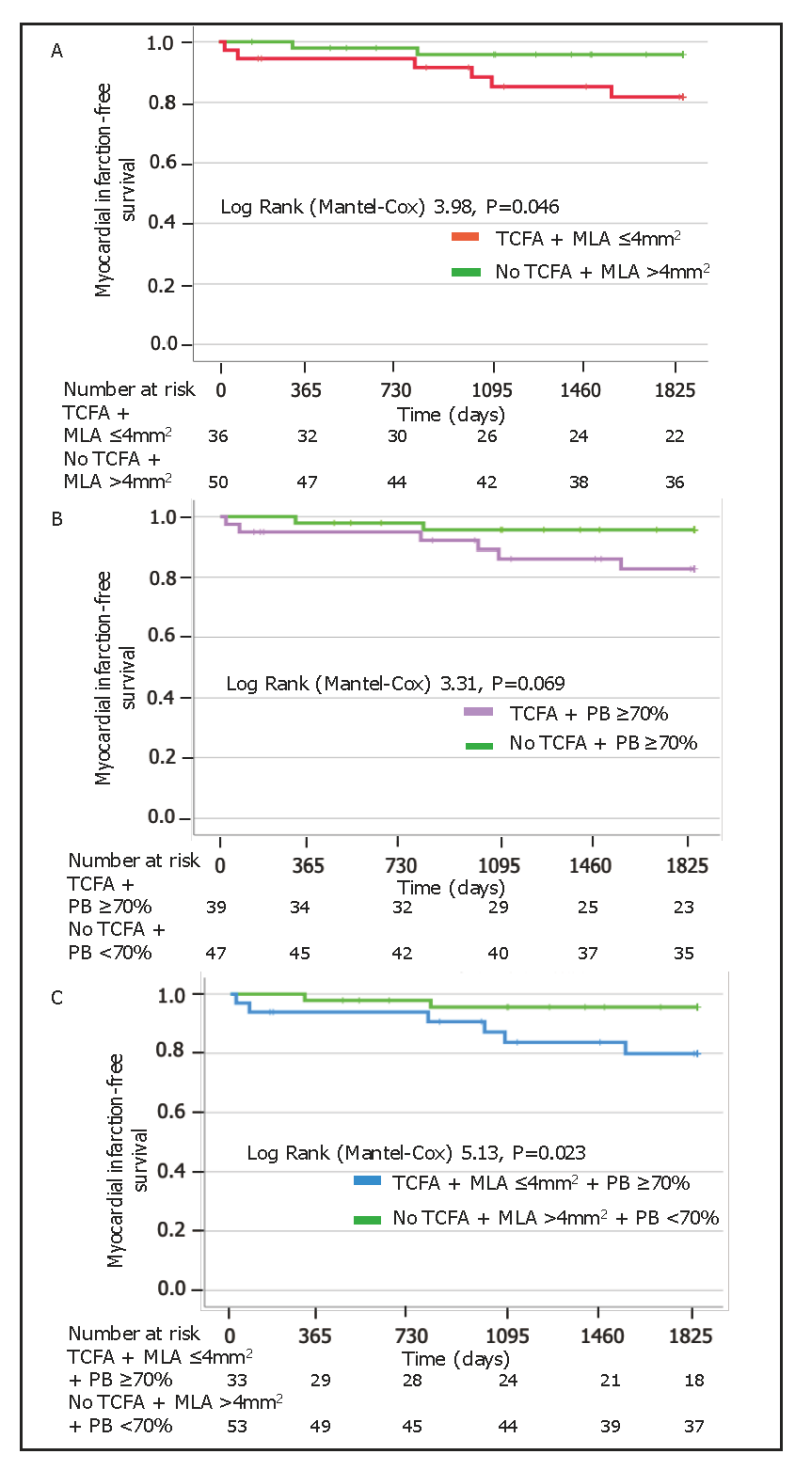
**Kaplan-Meier survival analysis**. MI-free survival in patients with TCFA and MLA ≤4 ${\mathrm{mm}}^{2}$ (A), TCFA and PB ≥70% (B) and combination of TCFA, MLA ≤4 ${\mathrm{mm}}^{2}$ and PB ≥70% (C). MI, myocardial infarction; MLA, minimal lumen area; PB, plaque burden; TCFA, thin-cap fibroatheroma.

The incidence of every component of the primary endpoint is shown in Table 1. Occurrence of MI was higher in patients with presence of TCFA, MLA ≤4 ${\mathrm{mm}}^{2}$ and PB ≥70% versus patients without the combination of the three characteristics (18.2% *vs.* 3.8%, *p* = 0.050) (Table 1 and Fig. 2). Kaplan-Meier curves demonstrated lower MI-free survival in patients with TCFA and MLA ≤4 ${\mathrm{mm}}^{2}$ (Log rank 3.98, *p* = 0.046) and in patients with the coexistence of three high-risk plaque characteristics (Log rank 5.13, *p* = 0.023) (Fig. 3A,C).

No other differences were found in terms of death, MI, and any revascularisation (**Supplementary Figs. 3–7** and Fig. 3B). The combination of TCFA, MLA ≤4 ${\mathrm{mm}}^{2}$ and PB ≥70% was correlated to the risk of MI (HR 5.23, 95% CI 1.05–25.9, *p* = 0.043) (Table 2 and Fig. 2). Univariable Cox regression analysis did not show other statistically significant associations (Table 2).

#### 3.3.2 Vessel-Level Analysis

The composite of MI and any revascularisation occurred in 8 (3.6%) vessels (Table 1). The incidence trend of adverse outcome was higher in lesions with high-risk plaque phenotypes than in those without these phenotypes despite the statistical significance was reached only in lesions with MLA ≤4 ${\mathrm{mm}}^{2}$ (8.0% *vs.* 1.3%, *p* = 0.018) (Table 1 and Fig. 2). The event-free survival was better in lesions with MLA >4 ${\mathrm{mm}}^{2}$ (Log rank 6.49, *p* = 0.011) and with the presence of the three high-risk plaque characteristics (Log rank 4.12, *p* = 0.042) (**Supplementary Figs. 8,9**). Lesions with MLA ≤4 ${\mathrm{mm}}^{2}$ were associated with an increased risk of TVF (HR 6.16, 95% CI 1.24–30.5, *p* = 0.026) (Table 2 and Fig. 2). No other statically significant associations were demonstrated (Table 2).

#### 3.3.3 Interleukin-6 and High-Risk Plaque Phenotypes Exploratory Data

Out of 86 patients, IL-6 levels were available in 69 patients (80.2%). The median value of IL-6 was 2.26 (IQR 1.38–3.47) ng/L (**Supplementary Table 1**). IL-6 levels were similar among the groups of patients with high-risk plaque characteristics (**Supplementary Table 3**). Patients with high-risk plaque characteristics showed increased levels of IL-6 (defined as ≥5 ng/L) despite lacking statistical significance (**Supplementary Table 5**). Level of IL-6 ≥5 ng/L was associated with the combination of TCFA, MLA ≤4 ${\mathrm{mm}}^{2}$ and PB ≥70% (OR 3.89, 95% CI 1.01–15.0, *p* = 0.048) (**Supplementary Table 6**).

## 4. Discussion

To the best of our knowledge, the current ICON1 study investigated for the first-time the correlation between plaque characteristics and clinical outcome at long-term follow-up in older patients with NSTEACS. The main findings are the following: (1) the presence of small lumen area (MLA ≤4 ${\mathrm{mm}}^{2}$) predicts adverse cardiovascular outcomes at 5-year follow-up; (2) MLA ≤4 ${\mathrm{mm}}^{2}$ in combination with TCFA and large PB increases the risk of MI 5-fold; (3) risk of TVF is associated with MLA ≤4 ${\mathrm{mm}}^{2}$; (4) TCFA and large PB did not demonstrate association with adverse clinical outcome on their own in our population.

The Providing Regional Observations to Study Predictors of Events in the Coronary Tree (PROSPECT) study demonstrated that lesions with TCFA, MLA ≤4 ${\mathrm{mm}}^{2}$ and PB ≥70% derived by VH-IVUS, were at high-risk to develop cardiovascular events at 3.4-year follow-up in patients with ACS [6]. The VH-IVUS in Vulnerable Atherosclerosis (VIVA) study and European Collaborative Project on Inflammation and Vascular Wall Remodelling in Atherosclerosis – Intravascular Ultrasound (ATHEROREMO-IVUS) study found similar results during follow-up [7, 8]. The results from the ATHEROREMO-IVUS study at 4.7 years of follow-up showed that small lumen area (≤4 ${\mathrm{mm}}^{2}$) was independently associated with the risk of MACE (1.49, 95% CI 1.07–2.08, *p* = 0.020), unlike TCFA (HR 1.27, 95% CI 0.91–1.77, *p* = 0.16) and PB ≥70% (1.33, 95% CI 0.92–1.93, *p* = 0.13) [24]. The prognostic role of MLA ≤4 ${\mathrm{mm}}^{2}$ was confirmed in our study in terms of MACE and TVF. In addition, the combination of small luminal area with large PB and TCFA increased the risk of MI 5-fold.

Many studies have demonstrated the effectiveness of statin therapy in the regression of PB and necrotic core [25]. In our study, 95.3% of patients were on statin therapy at discharge. Therefore, lipid-lowering therapy may have had a role in the plaque stabilization to prevent reinfarction. The lower effect of statin therapy on small luminal area could be correlated to the higher prevalence of dense calcium volume in plaques with MLA ≤4 ${\mathrm{mm}}^{2}$ compared to those with MLA >4 ${\mathrm{mm}}^{2}$ despite lacking statical significance (12.5 [IQR 8.30–20.9)] *vs.* 11.8 [IQR 5.53–18.2] *p* = 0.065) (**Supplementary Table 4**).

The comparison between our findings and results from the previous cited studies should consider some differences in terms of population and study endpoints. These studies investigated a population with a median age (PROSPECT: median age 58.1 years; VIVA: median age 63.1 years; ATHEROREMO-IVUS: mean age 61.6 years) lower than our cohort (median age 80.6 [IQR 78.2–83.2] years). Older patients demonstrated differences in plaque composition compared to young patients. A sub-analysis of the PROSPECT study showed greater plaque volume percentage of necrotic core (13.6% *vs.* 12.7%, *p* < 0.05), dense calcium (7.6% *vs.* 5.9%, *p* < 0.05) and lower percentage of fibrous tissue (58.2% *vs.* 60.1%, *p* < 0.05) in patients older than 64 years compared to patients aged <65 years [11]. In our cohort the mean plaque volume percentage of necrotic core (18.7 [±6.7] %) and the median of dense calcium (12.0 [IQR 7.04–19.3] %) appear higher than the findings of the PROSPECT sub-analysis [11]. The older age of our study population could explain these differences considering that atherosclerosis progresses with age. A study using OCT revealed increase in calcifications (28.6% in age 45–54 years *vs.* 54.0% in age ≥75 years, *p* < 0.001), lipid-rich plaque (33.6% in age 45–54 years *vs.* 49.4% in age ≥75 years) and decrease in median MLA (1.18 [IQR 0.80–1.84] in age 45–54 years *vs.* 0.99 [IQR 0.73–1.30] in age ≥75 years) with age [12].

These findings might be due to structural and functional changes in the vascular wall of the coronary circulation inducing endothelial dysfunction. Biological and genetic studies observed increasing expression of pro-oxidant and pro-inflammatory genes in older patients. Reactive oxygen species and inflammatory cells and cytokines lead to atherosclerosis progression [26]. In our study, we have explored the role of IL-6 as predictor of high-risk plaque characteristics. Patients with high-values of IL-6 (≥5 ng/L) had increased risk of high-risk plaques phenotype. Correlation between IL-6 and high-risk plaque has been already observed in patients with stable chest pain, where values of IL-6 >1.8 ng/L in combination with high-sensitivity cardiac troponin >1.5 ng/L were associated with high-risk plaque phenotype (OR 1.42, 95% CI: 1.06–1.90, *p* = 0.019 [27]. In our analysis we have considered a higher cut-off of IL-6 compared to the threshold used in the previous study (5 ng/L *vs.* 1.8 ng/L) because of the inclusion of older patients with ACS instead of stable patients with a young population (median age 60.2 ± 8.0 years). Increased values of circulating IL-6 have been considered a predictor factor of adverse clinical outcome in patients with ACS [21, 28]. Values higher than 3.97 ng/L showed increased risk of MACE including cardiovascular death, MI and stroke in patients with ACS (HR 1.57, 95% CI: 1.22–2.03, *p* = 0.0005) [28]. The association between high-risk characteristics, adverse cardiovascular outcomes and IL-6 supports its potential target role as therapeutic strategy in patients with acute ischaemic heart disease.

Risk of TVF, including MI and any revascularisation, increased in lesions with MLA ≤4 ${\mathrm{mm}}^{2}$ and not with TCFA and PB ≥70%. Lesions with small luminal area demonstrate increased shear stress [29]. It was supposed that high shear stress in the tight lesion could promote plaque erosion with endothelial cells exposure and subsequent thrombosis [12]. In addition, the combination of MLA ≤4 ${\mathrm{mm}}^{2}$ with TCFA and large PB contributes to the vulnerability of the plaque [27].

Identification of TCFA with VH-IVUS has demonstrated to be reliable compared to OCT despite reduced resolution [30, 31]. However, the technical limitation of IVUS could have a role in the lack of correlation between TCFA and adverse outcomes in our work.

Intravascular imaging allows the identification of high-risk plaque characteristics improving the prognostic risk evaluation even in older adults with ACS. Therefore, in the contemporary management of coronary artery disease, the role of intravascular imaging is not only to guide and optimise PCI, but also to improve the prognostic risk stratification of patients. This may be important in case of patients with multivessel disease. Further data are needed to validate our results in a large older population. In addition, the evaluation of high-risk coronary plaque using OCT may increase the robustness of the data.

### Strengths and Limitations

This study provides interesting and unique insights about the correlation between the long-term clinical outcome and plaque phenotypes in older patients with NSTEACS. Five-year follow-up data were completed for all patients and outcomes ascertainment from summary care and hospital records was robust. However, we are aware that this study has some limitations. First of all, the small sample size reduces the statistical power of survival analysis. The generalization of our results is limited by the selected older population included in the ICON1. Patients enrolled in the ICON1 came from UK centers and were referred for coronary angiography and PCI if needed. Therefore, they were considered sufficiently fit to undergo an invasive procedure. However, they are representative of the older population with an acceptable heath status due to improvements in medical care across various fields.

The vessel and patients-oriented clinical outcomes consisting of culprit and non-culprit related events were small. Therefore, we have not provided multivariable adjusted model for secondary endpoints because it would not have been statistically reliable. In our population, most of the patients died at home. Therefore, in those cases, the cause of the death cause was not available. Lack of data on cardiovascular death is a limitation of our work. Nevertheless, our study provides important insights into coronary artery plaque phenotype in older adults with NSTEACS and its association with long-term clinical outcomes.

## 5. Conclusions

Lesions with small luminal area with or without TCFA and large PB were associated with the occurrence of adverse clinical outcome at 5-year follow-up among older patients with NSTEACS. Combination of TCFA, MLA ≤4 ${\mathrm{mm}}^{2}$ and PB ≥70% was associated with high risk of MI.

## Data Availability

The data that support the findings of this study are available on request from the corresponding author upon reasonable request. The data are not publicly available due to privacy or ethical restriction.

## Supplementary Material

Supplementary material associated with this article can be found, in the online version, at https://doi.org/10.31083/j.rcm2505168.
