# Supplementary material for: Coronary Artery Plaque Phenotype and 5-Year Clinical Outcomes in Older Patients with Non-ST Elevation Acute Coronary Syndrome
Source: Rev Cardiovasc Med. 2024 May 14;25(5):168. doi: 10.31083/j.rcm2505168 (PMC11267207; doi:10.31083/j.rcm2505168)
Supplement: Supplementary file 1 [file 2153-8174-25-5-168-s1.docx]

Supplementary Material

1. Supplementary methods

The inclusion and exclusion criteria of the study to Improve Cardiovascular Outcomes in high-risk older patieNts with acute coronary syndrome (ICON1) are as follows.

Inclusion criteria:

- Patients aged ≥75 years old.
- Patients with a diagnosis of non-ST-elevation acute coronary syndrome.
- Patients planned for coronary angiogram or percutaneous coronary intervention.

Exclusion criteria:

- Patients with cardiogenic shock, primary arrhythmias, or significant valvular heart disease.
- Patients with malignancy with life expectancy <1 year.
- Patients with active infection (such as urinary infection, pneumonia or sepsis)
- Alternative diagnosis after coronary angiography (excluded after consent): pulmonary embolism, takotsubo cardiomyopathy, myocarditis, coronary vasospasm.
- Patients unable to consent (known dementia, language barrier, visual impairment, lack of capacity).

2. ****Supplemental tables****

**Supplementary Table 1. Baseline characteristics** **of patients**

| **Variables** | **Patients (n=86)** |
| --- | --- |
| **Demographic data** | |
| Age, years (IQR) | 80.6 (78.2-83.2) |
| BMI, Kg/m^2^ (IQR) | 26.8 (24.4-28.6) |
| Female, n (%) | 29 (33.7) |
| **Clinical scores** | |
| NYHA score ≥3, n (%) | 11 (12.8) |
| CCS score ≥3, n (%) | 10 (11.6) |
| Frailty (pre-frail + frail), n (%) | 61 (71.8) |
| GRACE score2.0, points (SD) | 128.8 (±18.4) |
| **Medical history** | |
| Arterial hypertension, n (%) | 53 (61.6) |
| Diabetes mellitus, n (%) | 19 (22.1) |
| Hyperlipidaemia, n (%) | 44 (51.2) |
| Family history of CAD, n (%) | 28 (33.3) |
| Smoker, n (%) | 33 (38.4) |
| Chronic kidney disease, n (%) | 15 (17.4) |
| Previous MI, n (%) | 16 (18.6) |
| Previous TIA/Stroke, n (%) | 13 (15.1) |
| COPD, n (%) | 15 (17.4) |
| **Blood tests** | |
| Haemoglobin, g/dL (SD) | 13.4 (±1.86) |
| White blood count, 10^^9^/L (SD) | 8.40 (±2.12) |
| Platelets, 10^^9^/L(IQR) | 230.0 (189.5-282.5) |
| Creatinine, mmol/L (SD) | 96.3 (±25.3) |
| eGFR, ml/min/1.73 m^2^ (IQR) | 54.2 (44.9-64.6) |
| Total cholesterol,* mml/L (SD) | 4.24 (±1.02) |
| Interleukin 6,^†^ ng/L (IQR) | 2.26 (1.38-3.47) |
| **Procedural and management data** | |
| NSTEMI, n (%) | 68 (79.1) |
| Single-vessel PCI, n (%) | 56 (65.1) |
| Multi-vessel PCI, n (%) | 27 (31.4) |
| Medical Management only, n (%) | 3 (3.5) |
| Left Main PCI, n (%) | 4 (4.7) |
| Left anterior descending artery PCI, n (%) | 52 (60.5) |
| Left circumflex artery PCI, n (%) | 25 (29.1) |
| Right coronary artery PCI, n (%) | 34 (39.5) |
| Number of stents, median (IQR) | 1 (1-2) |
| DES, n (%) | 79 (91.9) |
| Radial access, n (%) | 78 (90.6) |
| Contrast volume, mL (SD) | 201.1 (±74.5) |
| Peri-procedural complications, n (%) | 6 (7.0) |
| **Medical Therapy at the discharge** | |
| Aspirin, n (%) | 85 (98.8) |
| Clopidogrel, n (%) | 46 (53.5) |
| Ticagrelor, n (%) | 40 (46.5) |
| Statin therapy, n (%) | 82 (95.3) |
| ACEi/ARB, n (%) | 79 (91.9) |
| β-blocker, n (%) | 70 (81.4) |
| Ca^2+^-channel blocker, n (%) | 20 (23.3) |
| Long-acting nitrate, n (%) | 18 (20.9) |
| Nicorandil, n (%) | 7 (8.1) |
| Proton pump inhibitor, n (%) | 37 (43.0) |
| Warfarin, n (%) | 8 (9.3) |
| Vitamin D supplement, n (%) | 24 (27.9) |

* Total cholesterol data were available in 65/86 patients.

† Interleukin 6 data were available in 69/86 patients.

ACEi: angiotensin converting enzyme inhibitor; ARB: angiotensin II receptor blocker; BMI: body mass index; CAD: coronary artery disease; CCS: Canadian Cardiovascular Society; COPD: chronic obstructive pulmonary disease; DES: drug eluting stent; eGFR: estimated glomerular filtration rate; GRACE: Global Registry of Acute Coronary Events; IQR: interquartile range; MI: myocardial infarction; NSTEMI: non-ST elevation myocardial infarction; NYHA: New York Heart Association; PCI: percutaneous coronary intervention; SD: standard deviation; TIA: transient ischaemic attack.

**Supplementary Table 2. Baseline characteristics of vessels**

| **Variables** | **Vessels (n=225)** |
| --- | --- |
| Region of interest length, mm (IQR) | 40.5 (15.4-58.5) |
| MLA, mm^2^ (IQR) | 5.3 (3.5-8.0) |
| MLD, mm (IQR) | 2.6 (2.1-3.2) |
| Region of interest Plaque burden, % (IQR) | 65.4 (55.0-73.7) |
| Total frame number analysed, (IQR) | 86.5 (34.0-129.0) |
| FT % total plaque volume, (SD) | 53.7 (±12.4) |
| FF% total plaque volume, (IQR) | 12.2 (8.33-17.5) |
| NC% total plaque volume, (SD) | 18.7 (±6.7) |
| DC% total plaque volume, (IQR) | 12.0 (7.04-19.3) |
| Thick-cap fibroatheroma %, (IQR) | 36.2 (13.2-59.1) |
| Calcified thick-cap fibroatheroma %, (IQR) | 19.3 (5.04-39.3) |
| Thin-cap fibroatheroma %, (IQR) | 4.49 (0.00-13.6) |
| Calcified thin-cap fibroatheroma %, (IQR) | 2.99 (0.00-9.30) |
| Intimal medial thickening, n (%) | 5 (2.2) |
| Fibrotic plaque, n (%) | 7 (3.1) |
| Pathological intimal thickening, n (%) | 14 (6.2) |
| Fibrocalcific plaque, n (%) | 1 (0.4) |
| Fibroatheroma, n (%) | 33 (14.6) |
| Calcified fibroatheroma, n (%) | 114 (50.6) |
| Thin-cap fibroatheroma, n (%) | 81(36.0) |
| MLA ≤4mm^2^ | 75 (33.3) |
| PB ≥70% | 80 (35.5) |
| TCFA + MLA ≤4mm^2^ | 34 (15.1) |
| TCFA + PB ≥70% | 47 (20.8) |
| TCFA + MLA ≤4mm^2^ + PB ≥70% | 31 (13.7) |

DC: dense calcium; FF: fibro- fatty tissue; FT: fibrous tissue; IQR: interquartile range; MLA: minimal lumen area; MLD: minimal lumen diameter; NC: necrotic core; PB: plaque burden; SD: standard deviation; TCFA: thin-cap fibroatheroma

**Supplementary Table 3. Baseline characteristics of patients according to high-risk plaque characteristics**

| **High-risk plaque characteristics** | | | | | | | | | |
| --- | --- | --- | --- | --- | --- | --- | --- | --- | --- |
|  | **TCFA**  (n= 56) | **No TCFA**  (n= 30) | **P-value** | **MLA ≤4mm^2^**  (n= 51) | **MLA >4mm^2^**  (n= 35) | **P-value** | **PB ≥70%**  (n= 50) | **PB <70%**  (n= 36) | **P-value** |
| **Age, years (IQR)** | 81.2 (78.7-81.1) | 79.9 (77.2-81.8) | 0.138 | 80.7 (78.6-83.2) | 80.5 (77.2-82.7) | 0.463 | 81.2 (79.2-83.6) | 80.3 (77.3-82.5) | 0.090 |
| **BMI, Kg/m^2^ (IQR)** | 26.7 (24.6-29.0) | 27.0 (24.2-27.9) | 0.755 | 26.9 9 (24.2-28.6) | 26.7 (24.7-28.9) | 0.785 | 26.3 (24.2-28.2) | 27.5 (24.7-30.0) | 0.255 |
| **Female, n (%)** | 16 (28.6) | 13 (43.3) | 0.168 | 19 (37.3) | 10 (28.6) | 0.403 | 16 (32.0) | 13 (36.1) | 0.691 |
| **Arterial hypertension, n (%)** | 34 (60.7) | 19 (63.3) | 0.812 | 30 (58.8) | 23 (65.7) | 0.519 | 29 (58.0) | 24 (66.7) | 0.415 |
| **Diabetes mellitus, n (%)** | 14 (25.0) | 5 (16.7) | 0.375 | 9 (17.6) | 10 (28.6) | 0.230 | 10 (20.0) | 9 (25.0) | 0.581 |
| **Hyperlipidaemia, n (%)** | 31 (55.4) | 13 (43.3) | 0.288 | 24 (47.1) | 20 (57.1) | 0.358 | 25 (50.0) | 19 (52.8) | 0.799 |
| **Family history of CAD, n (%)** | 16 (29.1) | 12 (41.4) | 0.256 | 15 (30.0) | 13 (38.2) | 0.432 | 14 (29.2) | 14 (38.9) | 0.350 |
| **Smoking history, n (%)** | 3 (5.4) | 5 (16.7) | 0.085 | 3 (5.9) | 5 (14.3) | 0.187 | 1 (2.0) | 7 (19.4) | 0.006 |
| **Chronic kidney disease, n (%)** | 11 (19.6) | 4 (13.3) | 0.462 | 10 (19.6) | 5 (14.3) | 0.523 | 9 (18.0) | 6 (16.7) | 0.872 |
| **Previous MI, n (%)** | 10 (17.9) | 6 (20.0) | 0.808 | 9 (17.6) | 7 (20.0) | 0.783 | 9 (18.0) | 7 (19.4) | 0.865 |
| **Previous TIA/Stroke, n (%)** | 12 (21.4) | 1 (3.3) | 0.026 | 9 (17.6) | 4 (11.4) | 0.429 | 8 (16.0) | 5 (13.9) | 0.787 |
| **COPD, n (%)** | 8 (14.3) | 7 (23.3) | 0.292 | 9 (17.6) | 6 (17.1) | 0.952 | 5 (10.0) | 10 (27.8) | 0.032 |
| **Total cholesterol, * mml/L (SD)** | 4.05 (0.93) | 4.59 (1.10) | 0.043 | 4.21 (1.00) | 4.32 (1.06) | 0.540 | 4.10 (0.90) | 4.45 (1.15) | 0.241 |
| **Interleukin 6,^†^ ng/L (IQR)** | 2.29  (1.60-4.03) | 2.07  (1.11-3.42) | 0.169 | 2.21  (1.40-4.23) | 2.27  (1.21-3.12) | 0.612 | 2.44  (1.56-4.00) | 2.26  (1.12-2.94) | 0.195 |
| **Statin therapy, n (%)** | 54 (96.4) | 28 (93.3) | 0.516 | 47 (92.2) | 35 (100) | 0.090 | 47 (94.0) | 35 (97.2) | 0.484 |
|  | **TCFA+ MLA ≤4mm^2^**  (n= 36) | **No TCFA+ MLA >4mm^2^**  (n=50) | **P-value** | **TCFA+ PB ≥70%**  (n=39) | **No TCFA+ PB <70%**  (n=47) | **P-value** | **TCFA + MLA ≤4mm^2^ + PB ≥70%**  (n=33) | **No TCFA + MLA >4mm^2^+ PB <70%**  (n= 53) | **P-value** |
| **Age, years (IQR)** | 81.5 (79.3-84.1) | 80.4 (77.6-  82-0) | 0.145 | 81.8 (79.6-84.5) | 79.8 (77.2-  81-9) | 0.035 | 81.8 (79.4-84.2) | 79.8 (77.2-81.9) | 0.054 |
| **BMI, Kg/m^2^ (IQR)** | 26.5 (24.3-26.5) | 26.9 (24.4-  28-9) | 0.681 | 24.4 (24.2-28.6) | 27.2 (24.5-28.9) | 0.440 | 26.4 (24.1-28.3) | 27.2 (24.5-28.9) | 0.461 |
| **Female, n (%)** | 12 (33.3) | 17 (34.0) | 0.949 | 26 (66.7) | 31 (66.0) | 0.945 | 11 (33.3) | 16 (34.0) | 0.947 |
| **Arterial hypertension, n (%)** | 21 (58.3) | 32 (64.4) | 0.594 | 22 (56.4) | 31 (66.0) | 0.365 | 18 (54.5) | 31 (66.0) | 0.302 |
| **Diabetes mellitus, n (%)** | 7 (19.4) | 12 (24.0) | 0.615 | 7 (17.9) | 12 (25.5) | 0.399 | 7 (21.2) | 12 (25.5) | 0.655 |
| **Hyperlipidaemia, n (%)** | 17 (47.2) | 27 (54.0) | 0.535 | 18 (46.2) | 26 (55.3) | 0.397 | 16 (48.5) | 26 (55.3) | 0.547 |
| **Family history of CAD, n (%)** | 11 (30.6) | 17 (35.4) | 0.640 | 11 (28.9) | 17 (37.0) | 0.438 | 10 (30.3) | 17 (37.0) | 0.539 |
| **Smoking history, n (%)** | 1 (2.8) | 7 (14.0) | 0.077 | 1 (2.6) | 7 (14.9) | 0.050 | 1 (3) | 7 (14.9) | 0.082 |
| **Chronic kidney disease, n (%)** | 7 (19.4) | 8 (16.0) | 0.678 | 8 (20.5) | 7 (14.9) | 0.494 | 7 (21.2) | 7 (14.9) | 0.464 |
| **Previous MI, n (%)** | 5 (13.9) | 11 (22.0) | 0.340 | 7 (17.9) | 9 (19.1) | 0.887 | 4 (12.1) | 9 (12.1) | 0.402 |
| **Previous TIA/Stroke, n (%)** | 8 (22.2) | 5 (10.0) | 0.119 | 8 (20.5) | 5 (10.6) | 0.203 | 7 (58.3) | 5 (41.7) | 0.192 |
| **COPD, n (%)** | 4 (11.1) | 11 (22.0) | 0.189 | 5 (12.8) | 10 (21.3) | 0.304 | 4 (12.1) | 10 (21.3) | 0.289 |
| **Total cholesterol,* mml/L (SD)** | 3.85 (0.94) | 4.40 (1.05) | 0.049 | 4.02 (0.95) | 4.4 (1.04) | 0.078 | 4.00 (0.97) | 4.42 (1.05) | 0.066 |
| **Interleukin 6,^†^ ng/L (IQR)** | 2.20  (1.35-5.08) | 2.29 (1.40-3.34) | 0.850 | 2.47  (1.40-5.08) | 2.26  (1.27-3.29) | 0.334 | 2.21  (1.36-5.36) | 2.26  (1.39-3.28) | 0.591 |
| **Statin therapy, n (%)** | 34 (94.4) | 48 (96.0) | 0.735 | 37 (94.9) | 45 (95.7) | 0.848 | 31 (93.9) | 45 (95.7) | 0.715 |

* Total cholesterol data were available in 65/86 patients.

† Interleukin 6 data were available in 69/86 patients.

BMI: body mass index; CAD: coronary artery disease; COPD: chronic obstructive pulmonary disease; IQR: interquartile range; MI: myocardial infarction; MLA: minimal lumen area ; PB: plaque burden; SD: standard deviation; TCFA: thin-cap fibroatheroma; TIA: transient ischaemic attack.

**Supplementary Table 4. Baseline characteristics of vessels according to high-risk plaque characteristics**

| **High-risk plaque characteristics** | | | | | | | | | |
| --- | --- | --- | --- | --- | --- | --- | --- | --- | --- |
|  | **TCFA**  (n= 81) | **No TCFA**  (n= 144) | **P-value** | **MLA ≤4mm^2^**  (n= 75) | **MLA >4mm^2^**  (n= 150) | **P-value** | **PB ≥70%**  (n= 80) | **PB <70%**  (n=145) | **P-value** |
| FT % total plaque volume, (SD) | 50.0 (10.0) | 56.1 (13.2) | <0.001 | 52.3 (10.9) | 54.6 (13.0) | 0.168 | 48.7 (10.6) | 56.7 (12.4) | <0.001 |
| FF% total plaque volume, (IQR) | 11.0 (8.54-16.8) | 12.5 (8.18-18.45) | 0.056 | 12.0 (6.6-12.0) | 12.3 (8.3-18.9) | 0.368 | 12.9 (9.62-17.6) | 11.0 (7.30-17.4) | 0.039 |
| NC% total plaque volume, (SD) | 21.7  (5.4) | 16.9 (6.9) | <0.001 | 20.2 (5.70) | 19.0 (7.15) | 0.047 | 20.6 (5.72) | 17.8 (7.10) | 0.004 |
| DC% total plaque volume, (IQR) | 14.0 (8.42-21.9) | 9.95(4.92-17.9) | <0.001 | 12.5 (8.30-20.9) | 11.8 (5.53-18.2) | 0.065 | 14.0 (8.80-22.6) | 10.1 (5.95-16.6) | 0.002 |
|  | **TCFA+ MLA ≤4mm^2^**  (n= 34) | **No TCFA+ MLA >4mm^2^**  (n=191) | **P-value** | **TCFA+ PB ≥70%**  (n= 47) | **No TCFA+ PB <70%**  (n= 178) | **P-value** | **TCFA + MLA ≤4mm^2^ + PB ≥70%**  (n= 31) | **No TCFA + MLA >4mm^2^+ PB <70%**  (n= 194) | **P-value** |
| FT % total plaque volume, (SD) | 50.4 (9.91) | 54.5 (12.6) | 0.059 | 48.3 (9.90) | 55.3 (12.5) | <0.001 | 48.2 (10.0) | 54.5 (12.5) | 0.033 |
| FF% total plaque volume, (IQR) | 11.5 (8.50-14.0) | 12.2 (8.23-18.2) | 0.523 | 11.1 (9.30-16.4) | 12.3 (7.88-17.9) | 0.951 | 11.8(8.60-14.1) | 12.2 (8.13-17.9) | 0.790 |
| NC% total plaque volume, (SD) | 22.0 (5.60) | 18.2 (6.80) | 0.009 | 22.2 (5.18) | 17.9 (6.85) | <0.001 | 22.0(5.76) | 17.9 (6.85) | 0.011 |
| DC% total plaque volume, (IQR) | 11.3 (6.88-18.6) | 13.6 (8.11-21.3) | 0.090 | 16.5 (10.0-23.4) | 10.7 (6.57-17.5) | 0.002 | 13.8 (8.30-22.6) | 10.7 (6.57-17.5) | 0.082 |

IQR: interquartile range; SD: standard deviation; TCFA: thin-cap fibroatheroma; PB: plaque burden.

MLA: minimal lumen area; FT: fibrous tissue; FF: fibro- fatty tissue; NC: necrotic core; DC: dense calcium.

**Supplementary Table 5. Interleukin 6≥5ng/L stratified by high-risk plaque characteristics**

| **High-risk plaque characteristics** | | | | | | | | | |
| --- | --- | --- | --- | --- | --- | --- | --- | --- | --- |
|  | **TCFA** | **No TCFA** | **P-value** | **MLA ≤4mm^2^** | **MLA >4mm^2^** | **P-value** | **PB ≥70%** | **PB**  **<70%** | **P-value** |
| **~~Interleukin 6,~~**  **~~ng/L (IQR)~~** | ~~2.29~~  ~~(1.60-4.03)~~ | ~~2.07~~  ~~(1.11-3.42)~~ | ~~0.169~~ | ~~2.21~~  ~~(1.40-4.23)~~ | ~~2.27~~  ~~(1.21-3.12)~~ | ~~0.612~~ | ~~2.44~~  ~~(1.56-4.00)~~ | ~~2.26~~  ~~(1.12-2.94)~~ | ~~0.195~~ |
| **Interleukin 6**  **≥5ng/L, n (%)** | 9 (20.9) | 2 (7.7) | 0.188 | 8 (19.5) | 3 (10.7) | 0.505 | 7 (18.4) | 4 (12.9) | 0.743 |
|  | **TCFA+ MLA ≤4mm^2^** | **No TCFA+ MLA ≤4mm** | **P-value** | **TCFA+ PB ≥70%** | **No TCFA+ PB ≥70%** | **P-value** | **TCFA+ MLA ≤4mm^2^+ PB ≥70%** | **No TCFA+ MLA ≤4mm^2^+ PB≥ 70%** | **P-value** |
| **~~Interleukin 6, ng/L (IQR)~~** | ~~2.20~~  ~~(1.35-5.08)~~ | ~~2.29 (1.40-3.34)~~ | ~~0.850~~ | ~~2.47~~  ~~(1.40-5.08)~~ | ~~2.26~~  ~~(1.27-3.29)~~ | ~~0.334~~ | ~~2.21~~  ~~(1.36-5.36)~~ | ~~2.26~~  ~~(1.39-3.28)~~ | ~~0.591~~ |
| **Interleukin 6**  **≥5ng/L, n (%)** | 7 (25) | 4 (9.8) | 0.106 | 7 (25) | 4 (9.8) | 0.106 | 7 (28) | 4 (9.1) | 0.083 |

IQR: interquartile range; TCFA: thin-cap fibroatheroma; MLA: minimal lumen area; PB: plaque burden.

**Supplementary Table 6. Association between interleukin 6 ≥ 5 ng/L and high-risk plaque characteristics**

|  | **OR (95% CI)** | **P-value** |
| --- | --- | --- |
| **TCFA** | 3.17 (0.63-16.0) | 0.162 |
| **MLA ≤4mm2** | 2.02 (0.48-8.40) | 0.333 |
| **PB ≥70%** | 1.52 (0.40-5.7) | 0.535 |
| **TCFA+ MLA ≤4mm^2^** | 3.08 (0.80-11.7) | 0.100 |
| **TCFA+ PB ≥70%** | 3.08 (0.80-11.7) | 0.100 |
| **TCFA+ MLA ≤4 mm^2^+ PB ≥70%** | 3.89 (1.01-15.0) | 0.048 |

OR: odds ratio; TCFA: thin-cap fibroatheroma; MLA: minimal lumen area; PB: plaque burden.

**3. Supplemental Figures**

**
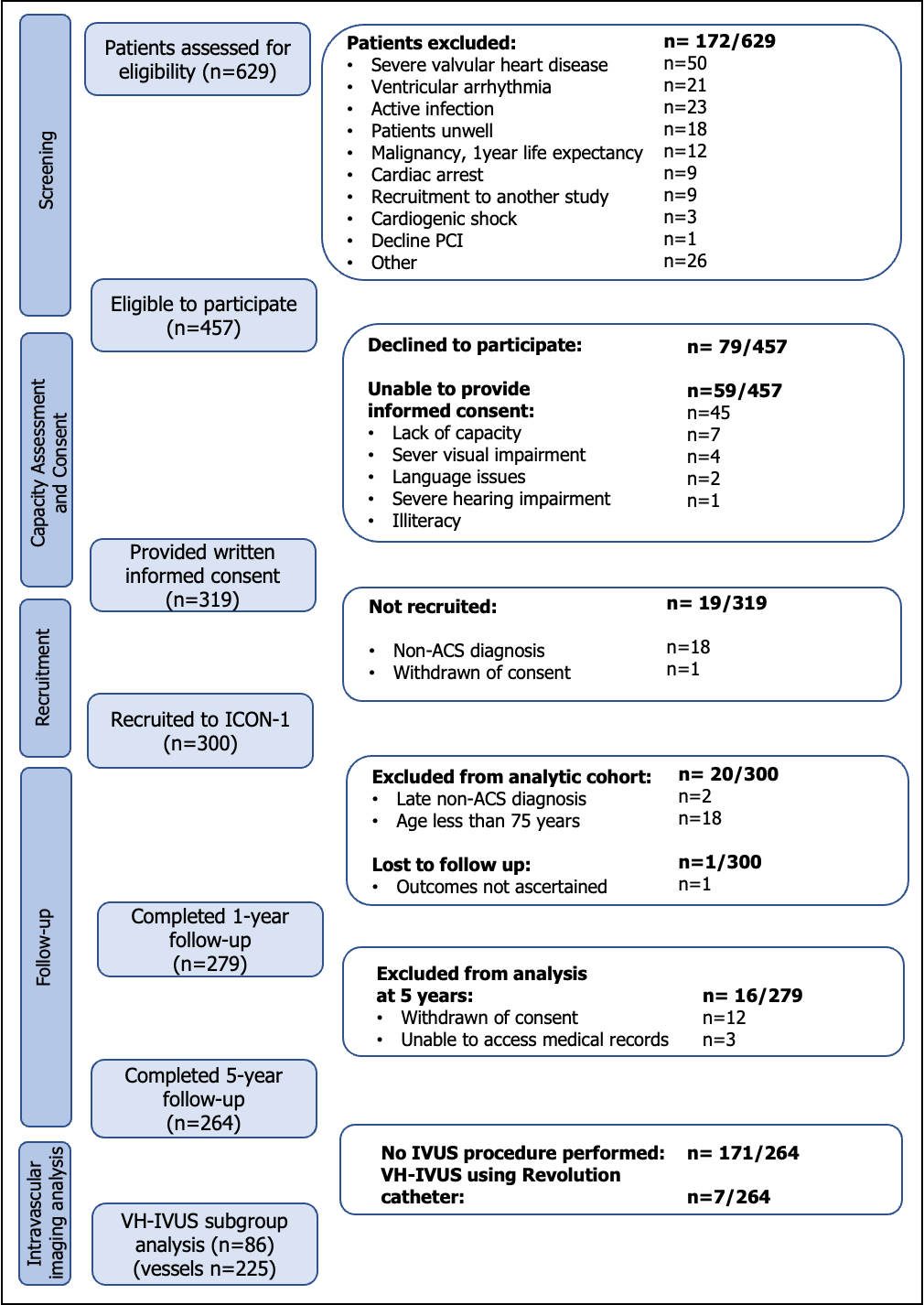
**

**Supplementary Fig. 1. Study flow chart.** ACS: acute coronary syndrome; ICON1: Improve Clinical Outcomes in high-risk patieNts with ACS; PCI: percutaneous coronary intervention; VH-IVUS: virtual-histology intravascular ultrasound


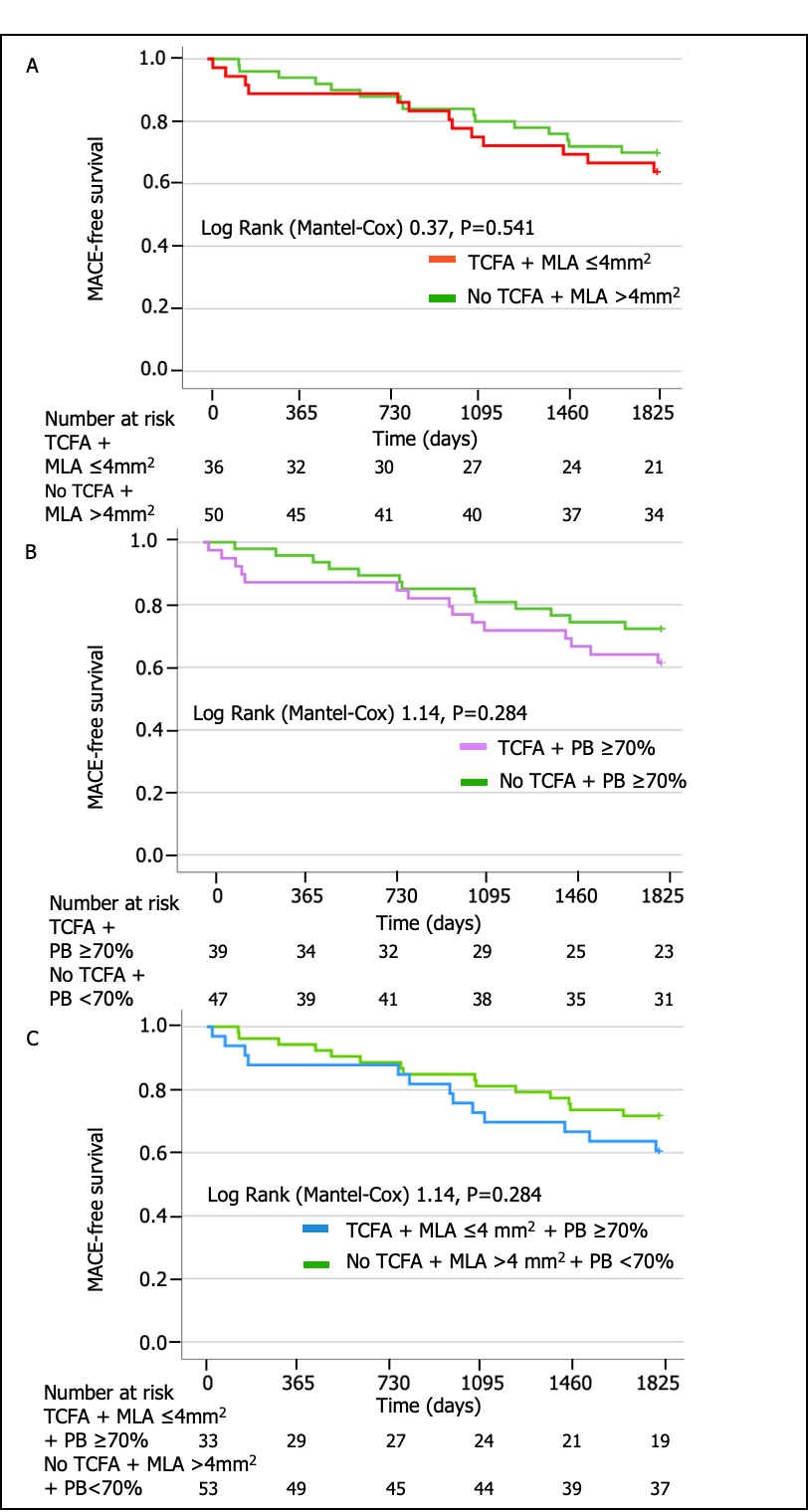


**Supplementary Fig. 2. Kaplan-Meier survival analysis.** MACE-free survival in patients with TCFA and MLA≤4mm2 (A), TCFA and PB ≥70% (B) and combination of TCFA, MLA≤4mm2 and PB ≥70% (C).

MACE: major adverse cardiovascular events; MLA: minimal lumen area; PB: plaque burden; TCFA: thin-cap fibroatheroma**.**


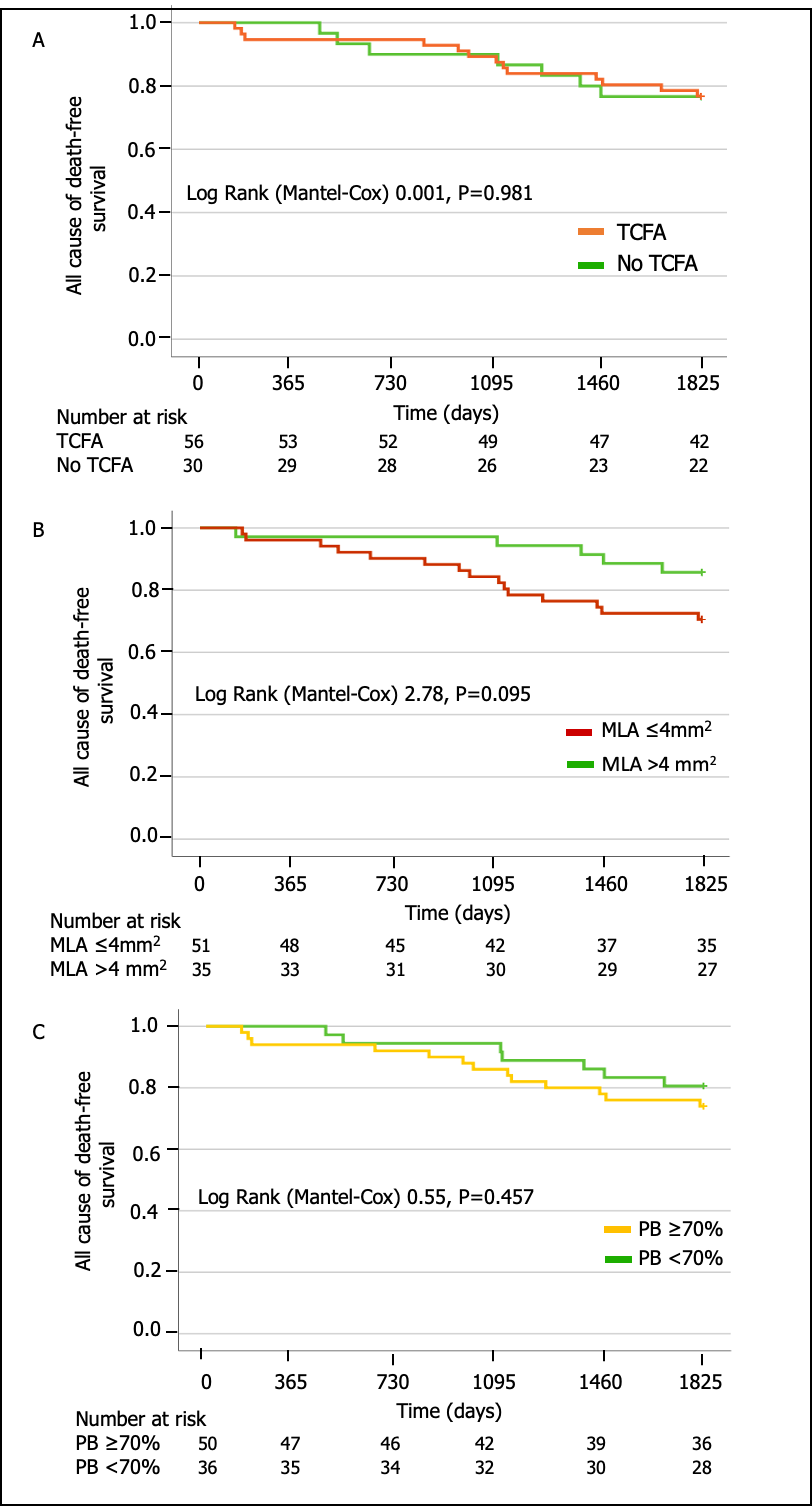


**Supplementary Fig. 3. Kaplan-Meier survival analysis.** All-cause mortality in patients with TCFA (A), MLA≤ 4mm^2^ (B) and PB ≥70% (C).

TCFA: thin-cap fibroatheroma; MLA: minimal lumen area; PB: plaque burden.


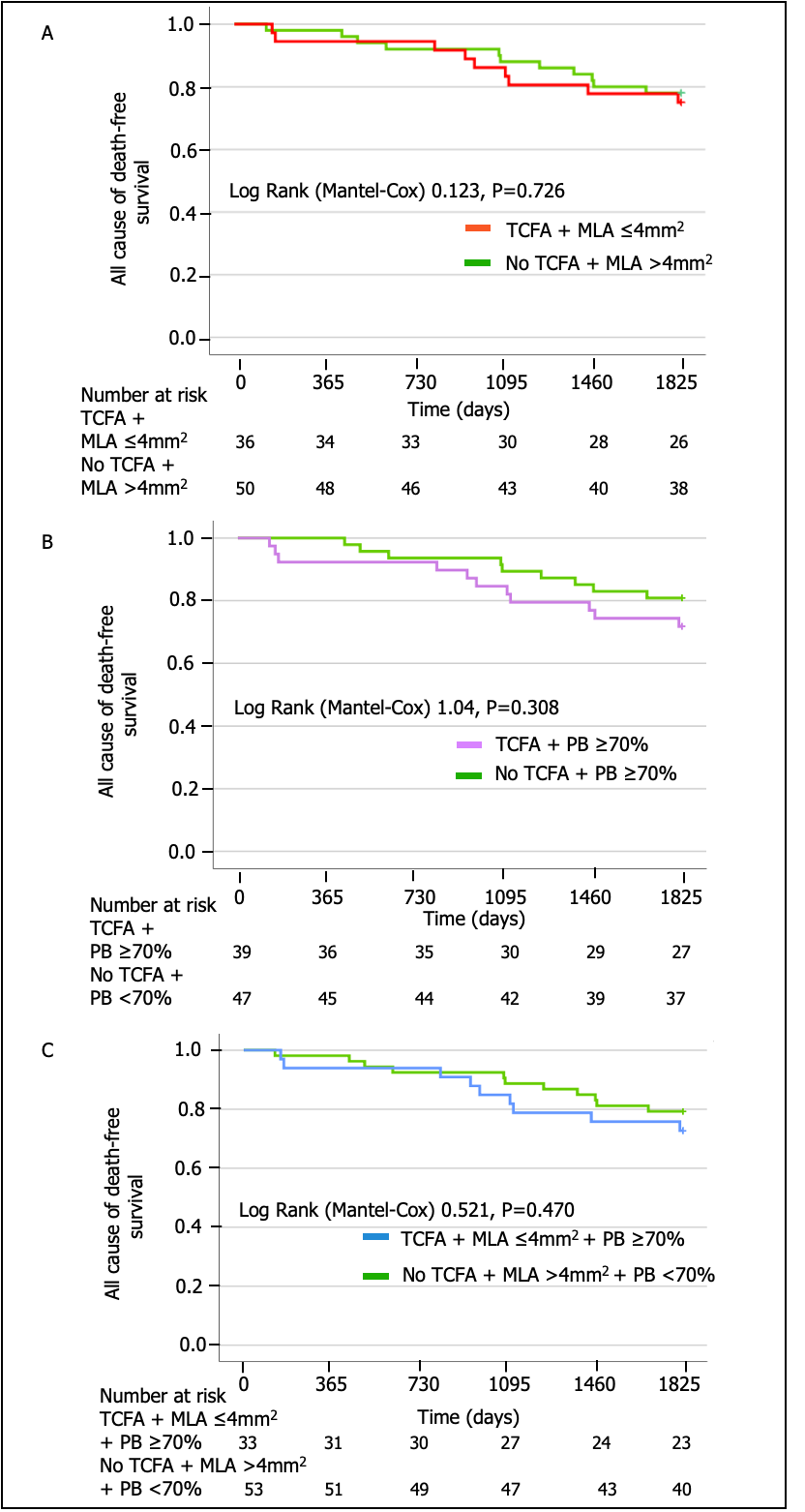


**Supplementary Fig. 4. Kaplan-Meier survival analysis.**

All-cause mortality in patients in patients with combination of TCFA and MLA ≤4mm^2^ (A), TCFA and PB ≥70% (B) and TCFA, MLA ≤4mm^2^ and PB ≥70% (C).

TCFA: thin-cap fibroatheroma; MLA: minimal lumen area; PB: plaque burden.

**
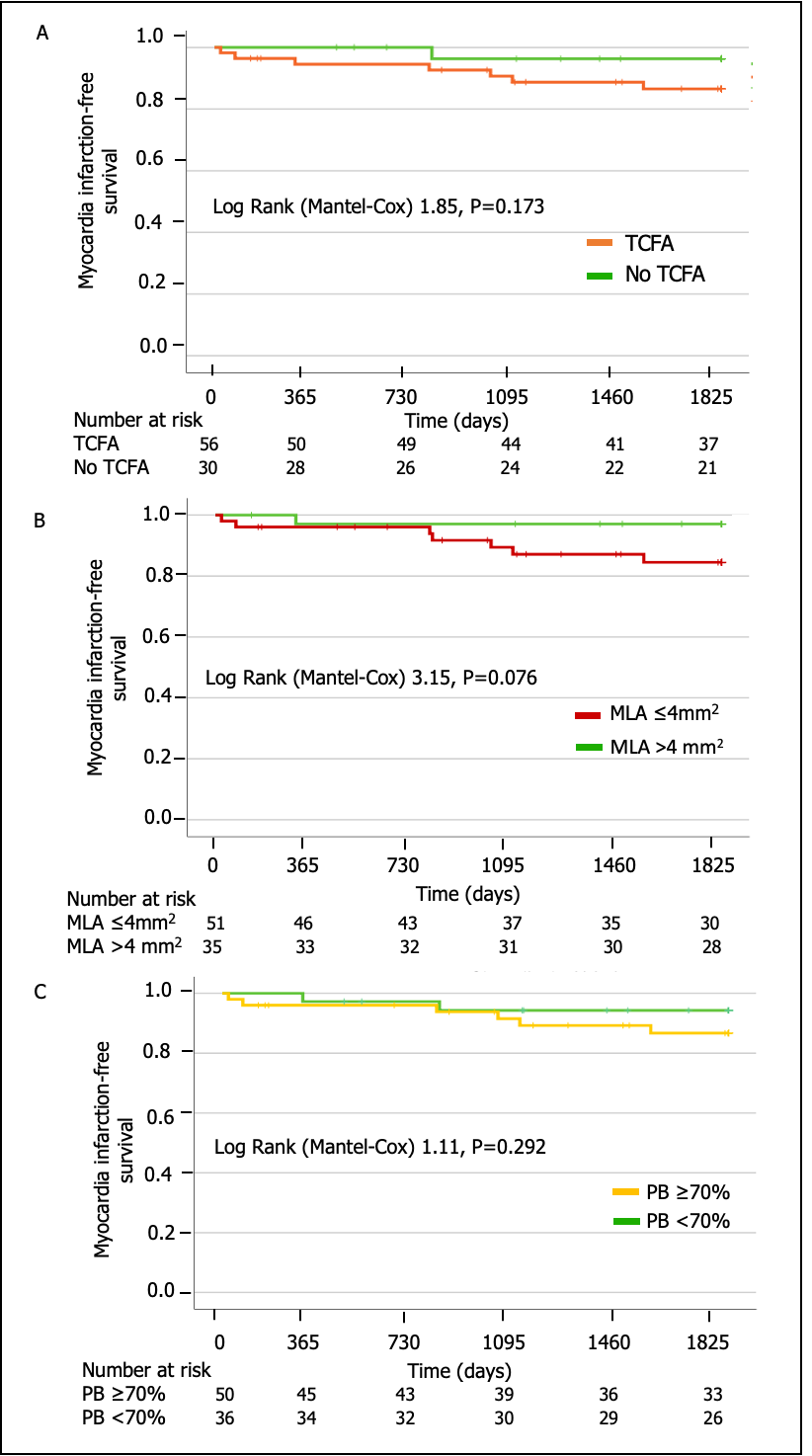
**

**Supplementary Fig. 5. Kaplan-Meier survival analysis.** MI-free survival in patients with TCFA (A), MLA ≤4mm^2^ (B) and PB ≥70% (C).

TCFA: thin-cap fibroatheroma; MLA: minimal lumen area; PB: plaque burden.


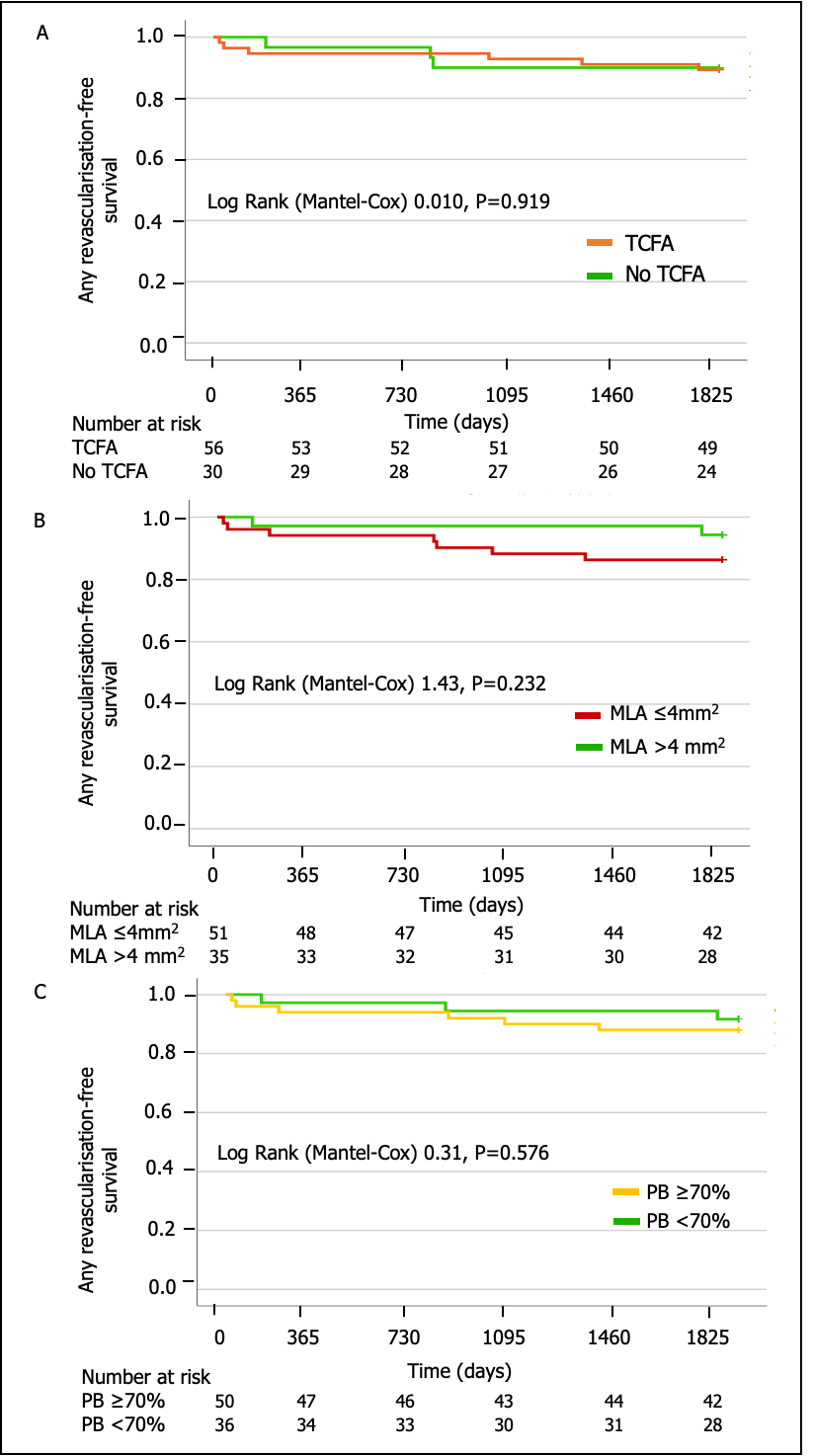


**Supplementary Fig. 6. Kaplan-Meier survival analysis.** Any revascularisation free survival in patients with TCFA (A), MLA ≤4mm^2^ (B) and PB ≥70% (C).

TCFA: thin-cap fibroatheroma; MLA: minimal lumen area; PB: plaque burden.


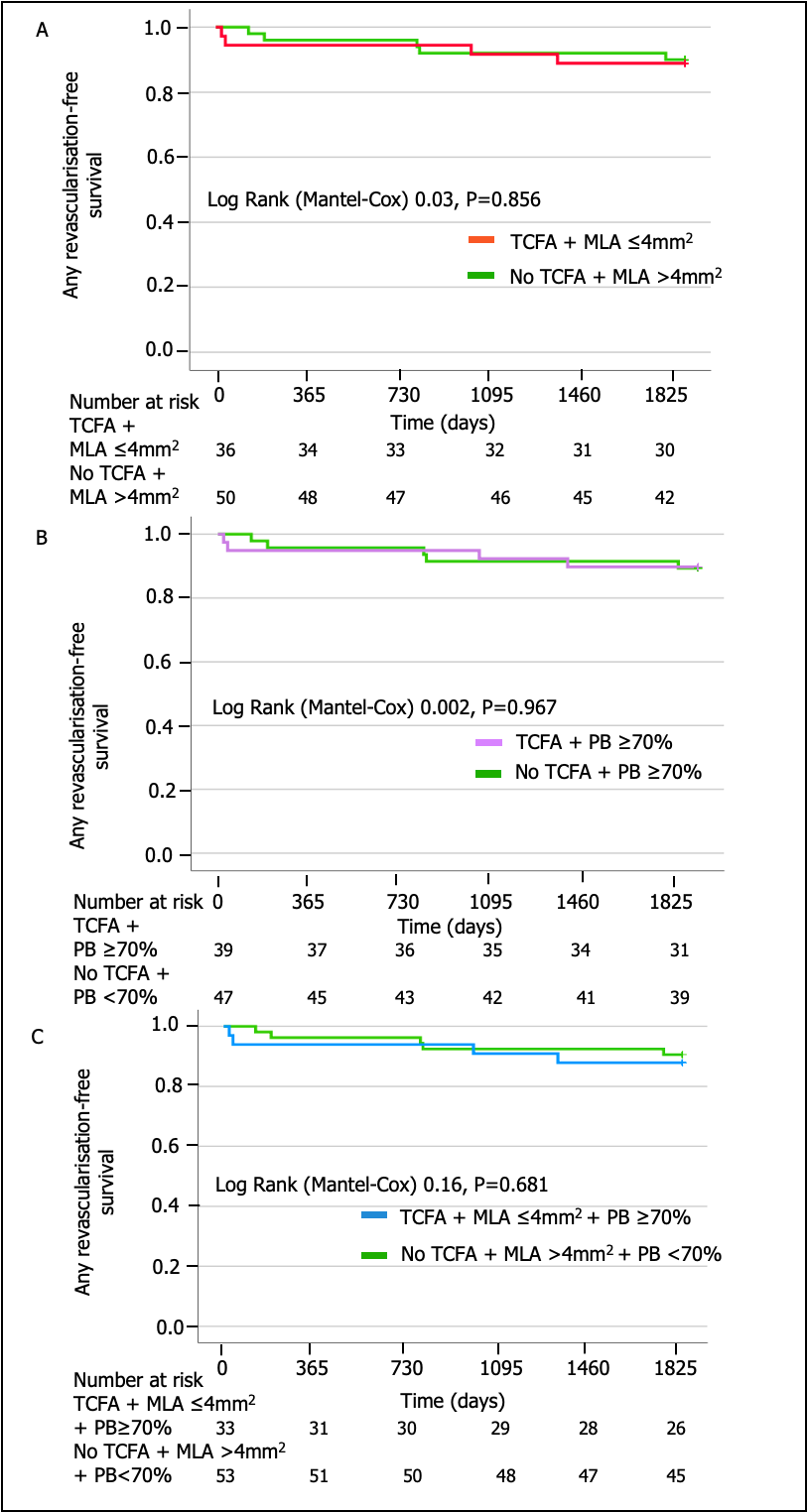


**Supplementary Fig. 7. Kaplan-Meier survival analysis.** Any revascularisation free survival in patients with combination of TCFA and MLA ≤4mm^2^ (A), TCFA and PB ≥70% (B) and TCFA, MLA ≤4mm^2^ and PB ≥70% (C).

TCFA: thin-cap fibroatheroma; MLA: minimal lumen area; PB: plaque burden.


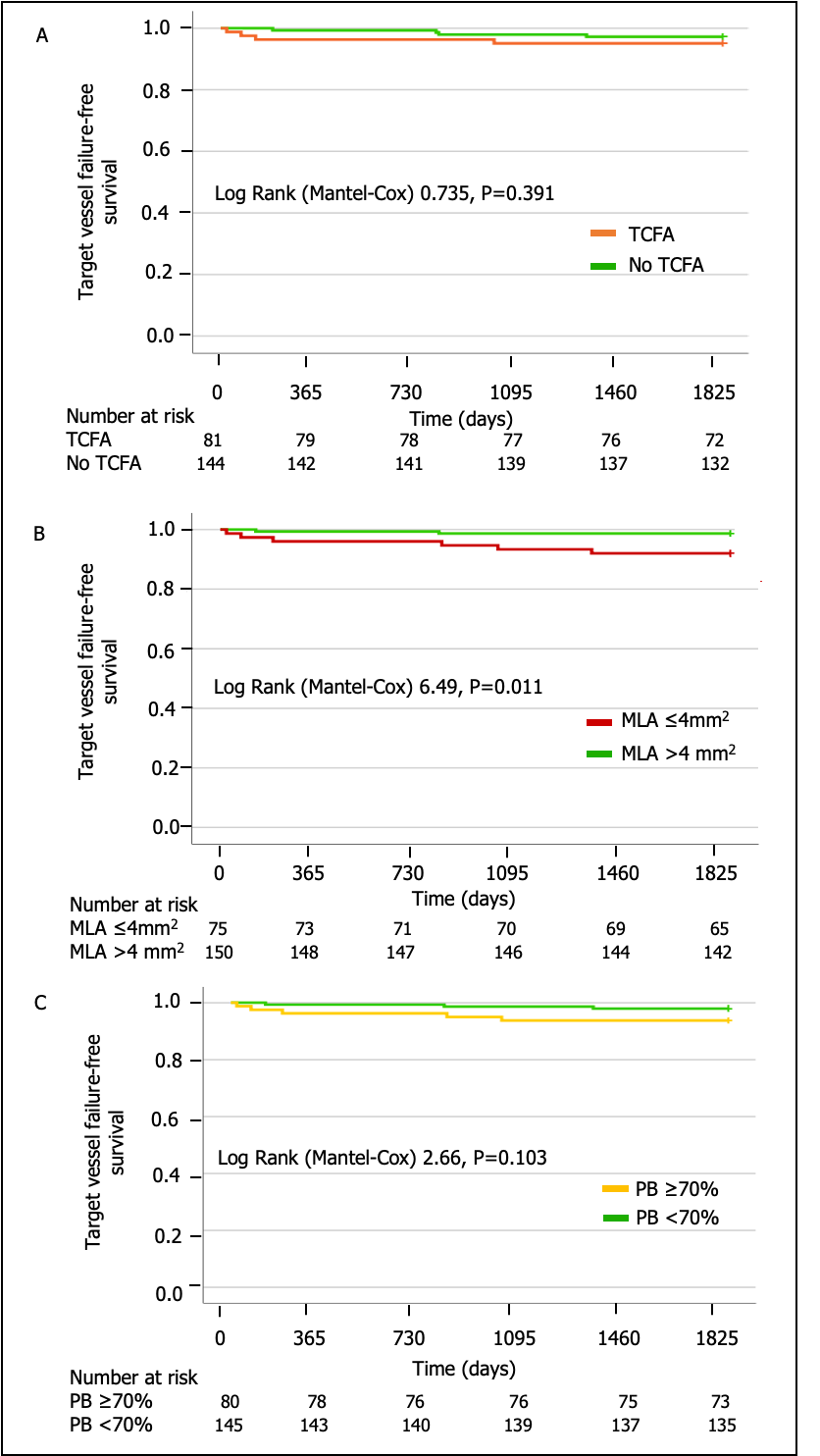


**Supplementary Fig. 8. Kaplan-Meier survival analysis.** Target vessel failure-free survival in lesions with TCFA (A), MLA ≤4mm^2^ (B) and PB ≥70% (C).

TCFA: thin-cap fibroatheroma; MLA: minimal lumen area; PB: plaque burden.


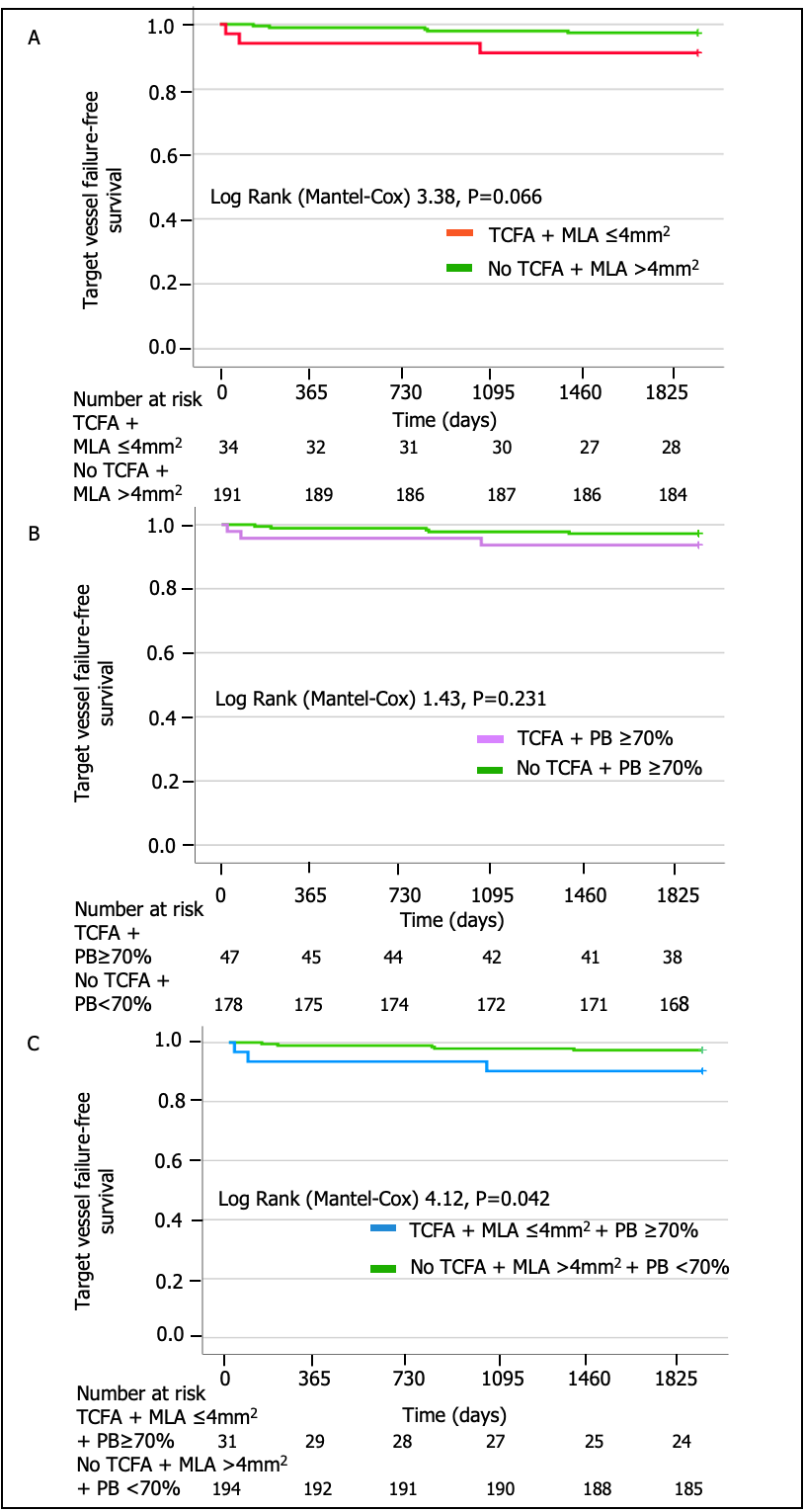


**Supplementary Fig. 9. Kaplan-Meier survival analysis.** Target vessel failure-free survival in lesions with a combination of TCFA and MLA ≤4mm^2^ (A), TCFA and PB ≥70% (B) and TCFA, MLA ≤4mm^2^ and PB ≥70% (C).

TCFA: thin-cap fibroatheroma; MLA: minimal lumen area; PB: plaque burden.

[1] Kunadian V, Neely RD, Sinclair H, et al. (2016) Study to Improve Cardiovascular Outcomes in high-risk older patieNts (ICON1) with acute coronary syndrome: study design and protocol of a prospective observational study. BMJ Open 6(8): e012091. 10.1136/bmjopen-2016-012091
